# Withdrawing biologics in non-systemic JIA: what matters to pediatric rheumatologists?

**DOI:** 10.1186/s12969-023-00845-4

**Published:** 2023-07-11

**Authors:** Janine A. van Til, Michelle M. A. Kip, Ellen J. H. Schatorjé, Gillian Currie, Marinka Twilt, Susanne M. Benseler, Joost F. Swart, Sebastiaan J. Vastert, Nico Wulffraat, Rae S. M. Yeung, C. G. M. (Karin) Groothuis-Oudshoorn, Sanne Warta, Deborah A. Marshall, Maarten J. IJzerman, on behalf of the UCAN CAN-DU, UCAN CURE consortia

**Affiliations:** 1 Department of Health Technology & Services Research, Faculty of Behavioural, Management and Social Sciences, Technical Medical Centre, University of Twente, P.O. Box 217, Enschede, AE The Netherlands; 2 Department of Pediatric Rheumatology, Division of Paediatrics, University Medical Center Utrecht, Wilhelmina Children’s Hospital, Utrecht, The Netherlands; 3 Department of Paediatric Rheumatology, St. Maartenskliniek, Nijmegen, the Netherlands; 4 Department of Paediatric Rheumatology and Immunology, Amalia Children’s Hospital, Radboud University Medical Center, Nijmegen, the Netherlands; 5 Alberta Children’s Hospital Research Institute, University of Calgary, Calgary, AB Canada; 6 Department of Community Health Sciences, Cumming School of Medicine, University of Calgary, Calgary, AB Canada; 7 Department of Paediatrics, Cumming School of Medicine, University of Calgary, Calgary, AB Canada; 8 Division of Rheumatology, Department of Pediatrics, Cumming School of Medicine, Alberta Children’s Hospital, University of Calgary, Calgary, AB Canada; 9 Faculty of Medicine, Utrecht University, Utrecht, The Netherlands; 10 European Reference Network RITA (rare immunodeficiency autoinflammatory and autoimmune diseases network), Utrecht, The Netherlands; 11 Division of Rheumatology, The Hospital for Sick Children, Department of Paediatrics, Immunology and Institute of Medical Science, University of Toronto, Toronto, Ontario Canada; 12 Department of Medicine, Cumming School of Medicine, University of Calgary, Calgary, Alberta Canada

**Keywords:** Juvenile Idiopathic Arthritis, Biologicals, Treatment withdrawal, Decision support tool, Clinical vignette study

## Abstract

**Objective:**

Approximately one third of children with JIA receive biologic therapy, but evidence on biologic therapy withdrawal is lacking. This study aims to increase our understanding of whether and when pediatric rheumatologists postpone a decision to withdraw biologic therapy in children with clinically inactive non-systemic JIA.

**Methods:**

A survey containing questions about background characteristics, treatment patterns, minimum treatment time with biologic therapy, and 16 different patient vignettes, was distributed among 83 pediatric rheumatologists in Canada and the Netherlands. For each vignette, respondents were asked whether they would withdraw biologic therapy at their minimum treatment time, and if not, how long they would continue biologic therapy. Statistical analysis included descriptive statistics, logistic and interval regression analysis.

**Results:**

Thirty-three pediatric rheumatologists completed the survey (40% response rate). Pediatric rheumatologists are most likely to postpone the decision to withdraw biologic therapy when the child and/or parents express a preference for continuation (OR 6.3; *p* < 0.001), in case of a flare in the current treatment period (OR 3.9; *p* = 0.001), and in case of uveitis in the current treatment period (OR 3.9; *p* < 0.001). On average, biologic therapy withdrawal is initiated 6.7 months later when the child or parent prefer to continue treatment.

**Conclusion:**

Patient’s and parents' preferences were the strongest driver of a decision to postpone biologic therapy withdrawal in children with clinically inactive non-systemic JIA and prolongs treatment duration. These findings highlight the potential benefit of a tool to support pediatric rheumatologists, patients and parents in decision making, and can help inform its design.

**Supplementary Information:**

The online version contains supplementary material available at 10.1186/s12969-023-00845-4.

## Introduction

Juvenile idiopathic arthritis (JIA) treatment has changed significantly with the introduction of biologic therapies. The availability of these biologic therapies, in addition to conventional therapies like methotrexate and intra-articular steroid injections, has increased the potential of attaining inactive disease and/or clinical remission in JIA, thereby preventing joint damage and long-term disability among these patients [1–4]. Currently, approximately one third of JIA patients receive biologic therapy during their treatment course [4, 5].

In contrast to guidelines regarding biologic therapy initiation, evidence and guidance on whether and how to withdraw biologic therapy from JIA patients with inactive disease and/or in clinical remission is lacking [2, 4, 6, 7]. Yet, timely withdrawal of biologic therapy is desirable to avoid prolonged exposure of the child to adverse effects like injection site reactions, as well as an increased risk of severe adverse events including infections requiring hospitalization, and malignancies [4, 8]. In addition, JIA medication, and in particular biologic therapy, is costly to families as well as to society [5, 9]. Decisions to withdraw treatment are inherently complex, with 3 in 4 patients flaring within 12 months after stopping biologic therapy [2, 10], and the risk of not being able to recapture inactive disease with the same medication [11, 12]. However, multiple studies have failed to yield conclusive evidence on clinical and biologic predictors for successful medication withdrawal in JIA, which further complicates these decisions [8]. International, evidence-based, consensus regarding the optimal timing of biologic therapy withdrawal in JIA after achieving sustained clinical remission is therefore warranted [4].

In previous studies, many factors were identified that influence the decision to withdraw medication in patients with JIA [8, 13–15]. These factors can be divided in characteristics of disease, such as the subtype of JIA, characteristics of treatment, such as the time to reach clinically inactive disease (CID), characteristics of the patient themselves, such as the patient’s preference to withdraw treatment, and contextual factors, such as accessibility of biologic therapies [8, 13, 14]. Three studies have previously investigated the relative importance of the different characteristics [8, 13, 15]. Time to reach CID and joint damage were consistently among the most important characteristics [8, 13, 15]. Other characteristics that were found relevant were therapy induced toxicity [8, 13], JIA subtype [8, 13], and patient and/or family preference for treatment withdrawal [8, 14]. In these studies, rating, ranking and best–worst scaling methods were used to determine the relative importance of the characteristics [8, 13, 15]. A drawback of these methods, is that they compare the relevance of different characteristics on a generic level, for one characteristic at a time. In clinical practice, every child will present a set of these characteristics, some of which would support biologic therapy withdrawal, for instance, the absence of joint damage, and some characteristics would support continuing biology therapy, such as a long time to reach CID.

The objective of this study is to increase our understanding of whether and when pediatric rheumatologists postpone a decision to withdraw biologic therapy in children with clinically inactive non-systemic JIA. To mimic the situation in which pediatric rheumatologists make decisions in current clinical practice, a vignette study design was used. In these vignettes, an experimental design was used to systematically vary patient-, disease-, and treatment characteristics to design different patient profiles. The first aim of this study is to assess the combined influence of patient-, disease- or treatment characteristics on the pediatric rheumatologists’ decision to postpone a decision to withdraw biologic therapy in children with clinically inactive non-systemic JIA. The second aim is to assess how long pediatric rheumatologists would treat children with clinically inactive non-systemic JIA with biologic therapy.

## Methods

### Data collection and respondent sample

This study involved a vignette study using an online survey format. The pediatric rheumatologists from Canada (*n* = 68) and the Netherlands (*n* = 15) who are part of the “Canada-Netherlands Personalized Medicine Network in Childhood Arthritis and Rheumatic Diseases” (UCAN CAN-DU)were invited to participate in this survey. First, an e-mail explaining the aim of the study was sent by the UCAN project management, followed by an invitation to participate which included the online link to the survey. Reminders were sent after four and 12 weeks. All responses were collected anonymously.

### Development of the descriptive framework for the clinical vignette study

The International League of Associations for Rheumatology (ILAR) defined seven distinct subtypes of JIA based on clinical and laboratory features [16]. Systemic JIA (10–20% of patients) is very distinct from the other six subtypes due to its systemic features and its different pathogenesis [17]. Therefore, the current study focuses on patients with non-systemic JIA.

Potential characteristics that influence a decision whether and when to withdraw biologic therapy were identified using a focus group with pediatric rheumatologists in Canada and one-on-one interviews with pediatric rheumatologists in the Netherlands. First, the focus groups in Canada yielded 14 patient-, disease- or treatment characteristics [14]. Second, the relative importance of these characteristics was determined in a Best Worst Scaling (BWS) study in Canada and the Netherlands [15]. Third, the interviews in the Netherlands were used to identify potential overlap and dependencies between the characteristics, that would limit their usability in a vignette study. A summary report of these interviews is included in Supplementary file 1. The findings in the interviews were verified with Canadian experts. Finally, the combined input from the focus groups, one-on-one interviews, and the BWS study was discussed by a team of clinical and methodological experts to support choices regarding design and content of the vignette study.

### Experimental design

The most important limiting factor regarding survey design was the number of clinical vignettes which could be answered by a single respondent. The ORTHOPLAN package in SPSS was used to calculate minimal orthogonal designs for scenarios where the clinical vignette study would include between 8–10 characteristics with two or three possible levels. The minimal orthogonal designs for these scenarios had between 12 and 27 questions. Pilot testing revealed that answering 16 vignettes was feasible for pediatric rheumatologists. After multiple rounds of discussion, the expert team selected the nine characteristics that were most relevant, which had no overlap, and which varied independently between patients (Supplementary Table 1). Characteristics for which it was important to distinguish three possible outcomes were the occurrence of flares, history of uveitis and prior treatment failure with biologics. All three situations could either not have occurred, have occurred in the current treatment period, or in a prior treatment period. The experimental design, which required 16 vignettes, is presented in Supplementary Table 2.

### Survey design

**Fig. 1:**
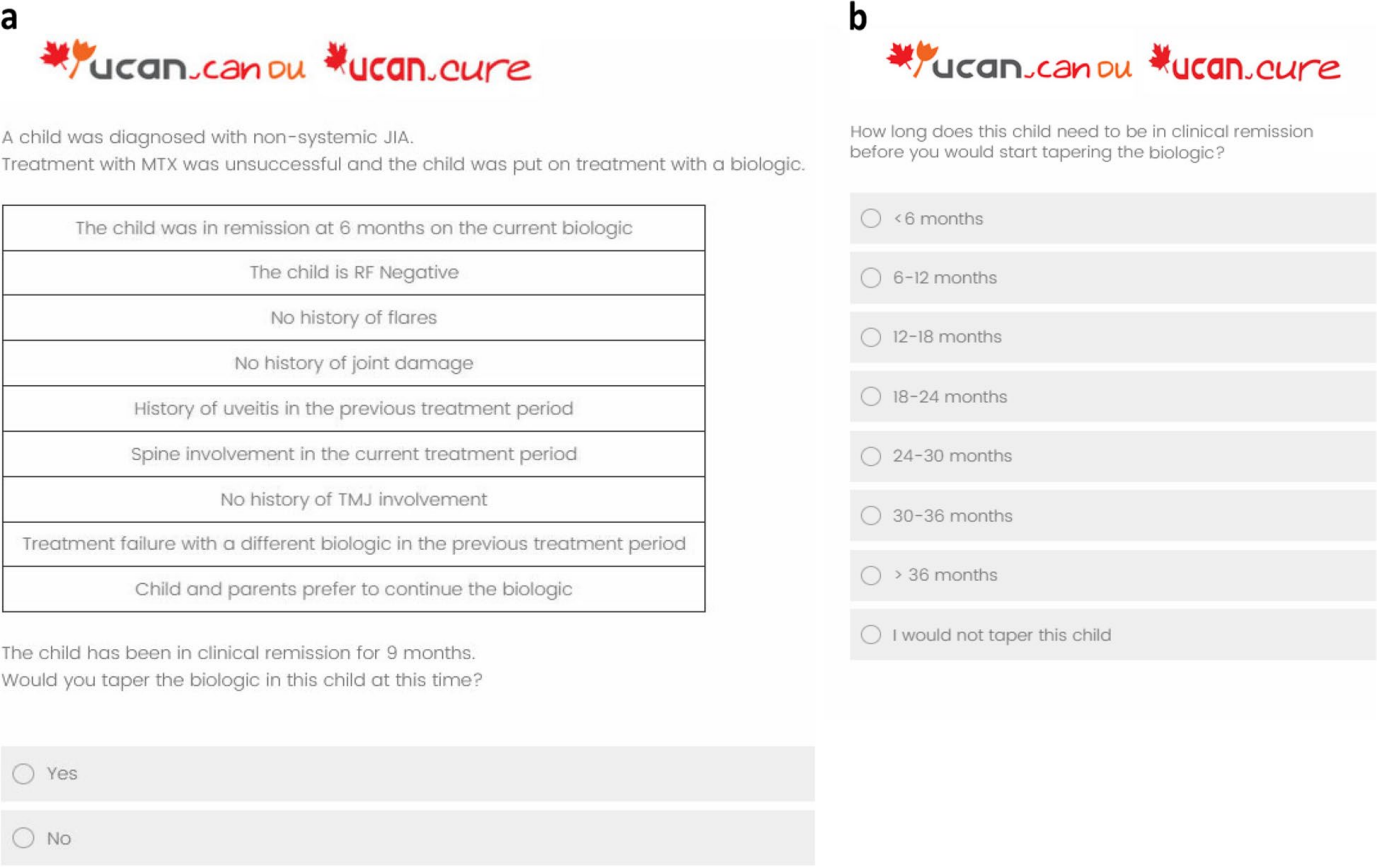
An example of the format of the clinical vignette question. In these vignettes, the study participant was first presented with a description of a child with non-systemic JIA (Juvenile Idiopathic Arthritis) (Fig. 1a). The nine patient-, disease- and treatment characteristics in the box were varied according to the experimental design. Then, the participant was asked whether they would taper the biologic in this child between 6 and 21 months after achieving clinical remission. The time in clinical remission was based on the participants own answer to an earlier question about minimal treatment time. If the participant answered negatively, they were directed to a second question (Fig. 1b) in which they were asked how long the child needs to be in clinical remission before the child is tapered. JIA = juvenile idiopathic arthritis; MTX = methotrexate; RF = rheumatoid factor; TMJ = temporomandibular joint

The survey contained three parts. The first part consisted of background questions on sex, age, country and region in which the respondent practices medicine, primary practice setting, the number of years clinical experience in general, the number of years clinical experience with children with JIA, and usual treatment with biologic therapy in children with JIA. This usual treatment included the biologic of first and second choice, and the time between the start of withdrawal and last dose of biologic therapy (immediate, 6, 9, 12, 15, 18, 21 months). The second part was the vignette study. Respondents were first presented with the most positive vignette of a child with JIA (response to biologic treatment within 6 months, RF-, no history of flares, inflammatory bowel disease (IBD), uveitis, no spine or temporomandibular joint (TMJ) involvement, no joint damage and a preference to taper biologic therapy) and asked how long they would treat this child with biologic therapy after achieving CID (6, 9, 12, 15, 18, 21 months, open answer possible). The answer to this question was considered the minimum time that the disease had to be clinically inactive before that respondent would consider to start biologic therapy withdrawal (time in CID). Then, the respondents were presented with 16 different vignette questions, in which the patient-, disease- and treatment characteristics were varied systematically across vignettes. After each vignette, the respondents were asked whether they would be willing to withdraw biologic therapy at the minimum time in CID as previously indicated, or whether they would postpone a decision to withdraw. If the respondents indicated they would postpone a decision to withdraw, they were then asked how long they would treat this child after achieving CID, which represents the total treatment time in CID, using intervals of six months (< 6 months; 6–12 months; 12–18 months; 18–24 months; 24–30 months; 30–36 months; I would not taper this child). The order of the vignettes was randomized over the respondents. An example of the format of the clinical vignette question is presented in Fig. 1.

The third part of the survey was a list of patient-, disease- and treatment characteristics which were not included in the vignette, but that were mentioned in literature. Respondents were asked to indicate how these characteristics influenced their withdrawal decisions (withdraw sooner, no influence, withdraw later than their minimum time in CID). Additionally, the Canadian pediatric rheumatologists were asked about the influence of restricted access to hospital and access to biologic therapy on their withdrawal decision. The survey can be found in Supplementary File 2.

### Statistical analysis

Statistical analysis was performed in R [18]. Descriptive analysis was used to calculate frequencies for interval and categorical scaled background characteristics. For the clinical vignette data, frequencies were used to describe variation across vignettes and between respondents. Logistic regression analysis was used to estimate the impact of patient-, disease-, and treatment characteristics (independent variables) on the decision to withdraw biologic therapy or to postpone this decision (dependent variable). The most positive outcome was used as the reference level for all characteristics, and 12 months was used as the reference category for time in CID. Interval regression analysis was used to estimate the impact of patient-, disease-, and treatment characteristics (independent variables) on total treatment duration (dependent variable). Treatment duration was coded in six-month intervals ranging from 0–6 months to 30–36 months. Minimum time in CID was included as a covariate in the analysis.

## Results

### Background characteristics

Complete responses were received from 24/68 (response rate 35.3%) pediatric rheumatologists from Canada and 9/15 (response rate 60.0%) pediatric rheumatologists from the Netherlands, resulting in an overall response rate of 40%. Background characteristics of the respondents are presented in Table 1. There were no major differences in sex, age, and clinical experience between pediatric rheumatologists from Canada and the Netherlands. All pediatric rheumatologists in the Netherlands work in academic hospitals, whereas in Canada some also work in community or solo practices. Pediatric rheumatologists from Canada see slightly more new patients and fewer existing patients each month compared to pediatric rheumatologists in the Netherlands.

**Table 1:** Background Characteristics of study participants

		**Canada**		**Netherlands**		**Total**	
		**n**	**%**	**n**	**%**	**n**	**%**
**Sample**		68	100%	15	100%	83	100%
**Response**		24	35%	9	60%	33	40%
**Sex**	**Male**	7	29%	3	33%	10	30%
	**Female**	17	71%	6	67%	23	70%
**Age**	**31–40**	4	17%	1	11%	5	15%
	**41–50**	11	46%	4	44%	15	45%
	** > 50**	9	38%	4	44%	13	39%
**Primary practice Setting**	**Academic setting, university based**	20	83%	9	100%	29	88%
	**Academic appointment but community based practice**	2	8%	0	0%	2	6%
	**Solo community based practice**	2	8%	0	0%	2	6%
**Experience**	** < 5 years**	4	17%	2	22%	6	18%
	**6–10 years**	4	17%	2	22%	6	18%
	**11–20 years**	9	38%	4	44%	13	39%
	**21–30 years**	7	29%	1	11%	8	24%
**Clinical work (% of FTE)**	** < 50%**	3	13%	1	11%	4	12%
	**50–75%**	11	46%	5	56%	16	48%
	** > 75%**	10	42%	3	33%	13	39%
**New patients each month (n)**	**1–3**	14	58%	8	89%	22	67%
	**4–6**	9	38%	1	11%	10	30%
**FU patients each month (n)**	**1–5**	4	17%	0	0%	4	12%
	**6–25**	10	42%	5	56%	15	45%
	**26–50**	9	38%	2	22%	11	33%
	** > 50**	1	4%	2	22%	3	9%
**Minimal treatment time after achieving CID**	**6 months**	0	0%	4	44%	4	12%
	**9 months**	1	4%	2	22%	3	9%
	**12 months**	12	50%	3	33%	15	45%
	**15 months**	2	8%	0	0%	2	6%
	**18 months**	3	13%	0	0%	3	9%
	**other**	6	25%	0	0%	6	18%
**Discontinuation strategy**	**immediately stop**	7	29%	1	11%	8	24%
	** < 6 months tapering**	10	42%	4	44%	14	42%
	**6–12 months tapering**	2	8%	1	11%	3	9%
	**18–24 months tapering**	1	4%	2	22%	3	9%
	**other**	4	17%	1	11%	5	15%

*CID* Clinically inactive disease, *FTE* Full-time equivalent, *FU* Follow-up

### Usual treatment strategy with biologic therapy

Biologic therapy of first choice is anti-TNFα as indicated by all participating pediatric rheumatologists in both Canada and the Netherlands. If this treatment fails, seven (10.3%) pediatric rheumatologists from Canada and five (33.3%) from the Netherlands prescribe a second biologic from the anti-TNFα category, while 16 (23.5%) pediatric rheumatologists from Canada and two (13.3%) from the Netherlands would switch to tocilizumab (anti-IL6). One rheumatologist from the Netherlands switches to CTLA-4 therapy, while one rheumatologist from the Netherlands and one from Canada replied that they need information about whether the patient either had primary treatment failure (i.e. non-response) or secondary treatment failure (i.e. loss of efficacy over time) on anti-TNFα before being able to decide which biologic to prescribe next.

### Clinical vignette study

The most commonly chosen time to biologic therapy withdrawal in a child without complications is 12 months after achieving CID (*n* = 15) (Table 1). Canadian respondents reported to initiate withdrawal of biologic therapy after 9 months of CID (*n* = 1), after 12 months (*n* = 12), after 15 months (*n* = 2), after 18 months (*n* = 3) or even after 21 months (*n* = 6), whereas pediatric rheumatologists in the Netherlands indicated to initiate withdrawal of biologic therapy in an uncomplicated child with JIA after 6 months (*n* = 4), after 9 months (*n* = 2), but no later than 12 months after achieving CID (*n* = 3). Duration of treatment with biologic therapy among pediatric rheumatologists in Canada is significantly longer than that of pediatric rheumatologists in the Netherlands (X^2^ = 17.5, *p*-value = 0.003). A detailed overview of the results is shown in Supplementary Tables 3–4.

Logistic regression analysis showed that patient-, disease-, and treatment characteristics that increase the odds that biologic therapy is continued are: 1) a preference of the child and/or parent to continue compared to a preference to withdraw biologic therapy (OR 6.34; *p* < 0.001), 2) a flare in the current treatment period compared to no history of flares (OR 3.90; *p* = 0.001), and 3) uveitis in the current treatment period compared to no history of uveitis (OR 3.86; *p* < 0.001). Pediatric rheumatologists who withdraw treatment after 9 months in CID in children without complications (OR 0.24; *p* < 0.001) and those who withdraw treatment after 21 months in CID (OR 0.16; *p* < 0.001) are more likely to withdraw treatment immediately in all of the clinical vignettes. This indicates that for them, the presence of complicating characteristics in the clinical vignettes is less of a reason to postpone a decision to withdraw than for pediatric rheumatologists who indicate an average treatment time of 12 months in CID. Odds ratios for all predictors with their significance levels are presented in Table 2.

**Table 2:** Influence of characteristics on the biologic therapy withdrawal decision. This table shows the influence of patient-, disease- and treatment-characteristics on their influence on the decision to withdraw biologic therapy (column 2–4) and total duration of treatment in clinically inactive disease (column 5–7). The first column lists the independent variables in the model. The dependent variable is the decision to postpone withdrawal of biologic therapy. The second column presents the odds ratio that indicates the impact of each characteristic on a decision to postpone withdrawal of biologic therapy with confidence interval and significance level. Reference category for each characteristic is the most positive outcome, e.g. no history of uveitis. Time in clinically inactive disease (CID) is included as a covariate. This is the minimal time a pediatric rheumatologist would treat a child with non-systemic JIA with the most positive profile. Reference category for Time in CID is 12 months. The fifth column indicates the influence of each of the characteristics on the total treatment duration, in months after clinical remission is achieved, with confidence interval and significance level

Withdrawal of biologic therapy in children with clinically inactive non-systemic JIA						
***Predictors***	***Odds Ratios***	***CI***	***p***	***Estimates***	***CI***	***p***
(Intercept)	0.19	0.07 – 0.43	** < 0.001**	16.56	11.40 – 21.71	** < 0.001**
Time in CID [6 months]	0.54	0.24 – 1.26	0.144	-17.48	-22.45 – -12.51	** < 0.001**
Time in CID [9 months]	0.24	0.10 – 0.56	**0.001**	-15.42	-21.00 – -9.84	** < 0.001**
Time in CID [15 months]	0.98	0.31 – 3.73	0.971	12.11	5.27 – 18.96	**0.001**
Time in CID [18 months]	0.59	0.24 – 1.57	0.268	6.13	0.43 – 11.83	**0.035**
Time in CID [21 months]	0.16	0.08 – 0.30	** < 0.001**	-0.11	-4.46 – 4.25	0.962
Response time [The child was in remission at 12 months on the current biologic]	1.74	0.96 – 3.18	0.067	0.15	-2.97 – 3.26	0.927
RF [The child is RF Positive]	1.77	1.01 – 3.12	**0.046**	7.81	4.70 – 10.92	** < 0.001**
Flares [History of flares in the previous treatment period]	1.50	0.81 – 2.94	0.207	1.06	-2.74 – 4.87	0.583
Flares [Flare in the current treatment period]	3.90	1.84 – 9.18	**0.001**	2.56	-1.25 – 6.38	0.188
Joint [Joint damage in the current treatment period]	2.39	1.34 – 4.47	**0.004**	3.27	0.16 – 6.38	**0.040**
Uveitis [Uveitis in the current treatment period, which is in remission]	3.86	1.91 – 8.33	** < 0.001**	3.29	-0.53 – 7.12	0.091
Uveitis [History of uveitis in the previous treatment period]	2.55	1.27 – 5.47	**0.011**	1.15	-2.66 – 4.97	0.553
Spine [Spine involvement in the current treatment period]	2.12	1.21 – 3.75	**0.009**	0.67	-2.44 – 3.78	0.673
TMJ [TMJ involvement in the current treatment period]	1.43	0.77 – 2.63	0.246	3.11	-0.01 – 6.22	0.051
Failure [Failure of a biologic in the current treatment period]	2.98	1.41 – 6.80	**0.006**	2.72	-1.09 – 6.53	0.162
Failure [Treatment failure with a different biologic in the previous treatment period]	2.32	1.21 – 4.54	**0.012**	4.36	0.54 – 8.17	**0.025**
Preference [Child and parents prefer to continue the biologic]	6.34	3.51 – 12.19	** < 0.001**	6.65	3.53 – 9.76	** < 0.001**
Log(scale)				2.88	2.82 – 2.95	** < 0.001**
Observations	528			528		
R^2^ Tjur	0.336			0.242		

*CID* Clinically inactive disease, *JIA* Juvenile idiopathic arthritis, *RF* Rheumatoid factor, *TMJ* Temporomandibular joint

The interval regression analysis showed that average treatment duration in CID is 16.6 months. Obviously, minimum treatment time in CID has the largest impact on total treatment time in CID. Treatment duration is significantly longer in a child with RF positive JIA compared to a child who is RF negative (7.8 months; CI = 4.7–10.9 months) and in a child that prefers to continue treatment (6.7 months; CI = 3.5–9.8 months) compared to a child who prefers withdrawal.

### Impact of other factors not included in the vignettes

Of the characteristics which were initially identified in the focus groups and interviews but that were excluded in the vignette study, hip involvement (61%), sacroiliac joint involvement (59%), and high disease activity (50%) were the three characteristics that were the most frequently chosen reasons to postpone the biologic therapy withdrawal decision beyond the minimum treatment time in CID. Pain at the injection site (32%) and fear of injections (38%) were the most frequently chosen reasons to withdraw biologic therapy sooner (Supplementary Table 5).

## Discussion

The results of this study indicate that the preference of a child and/or parent to continue biologic therapy is the most important reason to postpone a decision to withdraw biologic therapy in children with clinically inactive non-systemic JIA, and also results in the longest increase in total treatment time with biologic therapy after the child has reached CID. The only study that previously investigated the influence of treatment preferences on decision making was the study of Horton et al., 2017 [8]. The high importance of patient and/or parent preferences likely reflect the current complexity in decision making, in which there is no clear evidence regarding the influence of patient-, disease- or treatment characteristics on successful biologic therapy withdrawal. This finding also emphasizes the need to involve children and their parents in the decision making process, to inform them about the factors that may influence successful withdrawal, but also about the high uncertainty in predicting this outcome. Deciding whether and when to withdraw biologic therapy involves a trade-off between benefits and possible harms. A clear benefit of withdrawal is that it removes the burden of treatment for children, and results in lower costs of treatment of JIA for the parents and for society. The harms if withdrawal fails are a disease flare, the subsequent need to restart and potentially intensify biologic therapy, and the associated clinical and emotional burden to patients [12, 19]. Especially in Canada, restarting biologic therapy is associated with possible hurdles, primarily related to access to the medication and reimbursement barriers [20], and although to a lesser extent, limited access to health care especially in the winter months.

Our study confirmed the considerable variation between pediatric rheumatologists about medication withdrawal decisions in patients with JIA which was found in other studies [2]. In addition, there is a clear effect of country in this study, with longer minimum treatment time with biologic therapy in uncomplicated children with non-systematic JIA in CID among pediatric rheumatologists from Canada compared to the Netherlands (Supplementary Tables 6 and 7). In Canada, there is stricter regulation of access to and reimbursement of biologic therapy and more limited access to hospitals compared to the Netherlands. This likely increases the time between symptom onset and start of biologic therapy in Canada compared with the Netherlands, which is an indicator for poorer outcomes, and might make pediatric rheumatologists more hesitant to withdraw treatment in the first place. Second, it is more difficult to restart biologic therapy once withdrawn. However, the results of our study also indicate that pediatric rheumatologists who indicate a longer minimum treatment time on biologic therapy in children without complications are more likely to withdraw biologic therapy in children with complications. Also, pediatric rheumatologists in Canada had more abrupt treatment withdrawal strategies compared to pediatric rheumatologists in the Netherlands.

Other treatment choices are in line with previous research. The average minimum treatment time of 12 months is in line with other literature [8], which strengthens the external validity of the study findings. The indicated biologic of first choice in both Canada and the Netherlands, i.e. TNF-α inhibitors, is in line with current treatment guidelines [7, 21]. Previously, adalimumab was reported to be the most commonly prescribed TNF- α biologic in the Netherlands [22], whereas in Canada etanercept was most commonly prescribed [23].

### Implications for practice

Currently, JIA treatment guidelines state that recommendations on treatment withdrawal in non-systemic JIA patients cannot be provided due to the lack of available evidence, specifically a lack of evidence on biomarker-based withdrawal approaches [7, 21]. Evidence on such biomarker-based approaches is however emerging and promising [24, 25]. Previous research among patients with rheumatoid arthritis found that the discussion of medication withdrawal “should take place with the identification of patients’ priorities and in the context of their personal disease experiences” [26]. In this project, we aim to complement a biomarker-based approach with clinical judgement and with preferences of patients and/or parents to improve the quality of care for JIA. The results of this study will be used to inform a decision support tool, that is currently being developed. The tool will use a combination of multi-criteria decision analysis and prediction models to inform pediatric rheumatologists, children with JIA and parents about the relative influence of patient-, disease- and treatment characteristics, based on the choices of a sample of peers, on treatment choices regarding withdrawal of biologic therapy. With this tool we aim to promote evidence and preference based shared decision making between parents, children and pediatric rheumatologists [27].

### Strengths and limitations

The first strength of this study is the use of a vignette design, in which different characteristics are simultaneously presented in a single patient profile, compared to a preference elicitation method in which the impact of *individual* characteristics is investigated one at a time. This is a better reflection of clinical practice [28]. The use of an experimental design to develop the clinical vignettes enables to systematically vary the patient population, and thereby to also assess the less common cases in these clinical vignettes [28].

However, there are also some limitations to our study. First, not all patient-, disease- and treatment characteristics of JIA were included in the clinical vignette. These characteristics included amongst others hip involvement, sacroiliac joint involvement and high disease activity. However, including all characteristics is impossible as this would have increased both the information in the clinical vignette and the number of clinical vignettes that had to be answered beyond what was regarded feasible.

Second, in the vignettes, there was no distinction between children that were on combination therapy of methotrexate and a biologic, or on biologic monotherapy. Some pediatric rheumatologists commented that such combination therapy would have influenced their withdrawal decisions. These could be threats to the external validity of the study findings.

Third, the response rate in this study was 60.0% among the pediatric rheumatologists in the Netherlands, but only 35.3% in Canada. Similar response rates have however been described in other web-based surveys among medical specialists in Canada [29]. In addition, this questionnaire was distributed during the Covid pandemic, which was found to have a negative impact on response rates [30].

A final limitation of our study is that the smaller sample size in the Netherlands, due to the limited number of pediatric rheumatologists working there, did not allow us to test for differences between the two countries.

## Conclusions

Patient’s and parents' preferences were the strongest driver of a decision to postpone biologic therapy withdrawal in children with clinically inactive non-systemic JIA. Treatment duration is increased with about six months when patients and parents prefer to continue treatment. There is a large variation in minimum treatment time between pediatric rheumatologists, which is partly explained by the country of residence which may reflect different underlying medication access issues. These findings emphasize the need, and inform the design of, a tool to support pediatric rheumatologists, patients and parents in decision making.

## Supplementary Information

**Additional file 1. **Results of interviews with pediatric rheumatologists.

**Additional file 2:** Survey Instrument.

**Additional file 3:** **Supplementary Table 1. **Characteristics and levels inluded in the clinical vignettes.

**Additional file 4:** **Supplementary Table 2. **The experimental design of the 16 clinical vignettes.

**Additional file 5:** **Supplementary Table 3.** Descriptive data for each of the 16 clinical vignettes.

**Additional file 6:** **Supplementary Table 4.** Withdrawal of biologic therapy at each time interval in response to the clinical vignettes.

**Additional file 7:** **Supplementary Table 5.** Perceived influence of other disease characteristics on the withdrawal decision.

**Additional file 8:** **Supplementary Table 6.** Decision to continue biologic therapy dependent upon country. **Supplementary Table 7.** Treatment duration with biologic therapy dependent upon country.

## Data Availability

The data underlying this article cannot be shared publicly for the privacy of individuals that participated in the study. The data will be shared on reasonable request to the corresponding author.

## Abbreviations

BWS: Best Worst Scaling
CID: Clinically inactive disease
IBD: Inflammatory bowel disease
ILAR: International League of Associations for Rheumatology
JIA: Juvenile idiopathic arthritis
OR: Odds ratio
TMJ: Temporomandibular joint
UCAN CAN-DU: Canada-Netherlands Personalized Medicine Network in Childhood Arthritis and Rheumatic Diseases
UCAN CURE: Precision Decisions for Childhood Arthritis
