# Supplementary material for: Withdrawing biologics in non-systemic JIA: what matters to pediatric rheumatologists?
Source: Pediatr Rheumatol Online J. 2023 Jul 11;21:69. doi: 10.1186/s12969-023-00845-4 (PMC10337208; doi:10.1186/s12969-023-00845-4)
Supplement: Supplementary file 1 — Additional file 1. Results of interviews with pediatric rheumatologists. [file 12969_2023_845_MOESM1_ESM.pdf]

Juvenile Idiopathic Arthritis: How do pediatric rheumatologists decide when to stop or taper a biological?

Authors: Janine van Til; Maarten IJzerman; Michelle Kip

Project team: Deborah Marshall; Gillian R. Currie; Marinka Twilt

Background of this report

This report describes the result of the interviews which were performed with 12 Dutch pediatric rheumatologists. The aim of the interviews was to understand the current decision process regarding the decision to taper or stop a biological in children with juvenile idiopathic arthritis. Based on the interviews, a first version of a multi-criteria decision model was built, which is presented at the end of this report.

The primary aim of this report is to inform the participants of the interviews on the interview outcomes.

Project objectives:

- Develop a decision support tool for clinicians to assist in the decision to stop treatment with biologics in children with juvenile idiopathic arthritis.
- Adapt the tool to Canadian and Dutch context
- Pilot test the tool among Dutch and Canadian pediatric rheumatologists
- Implement the tool in an online, stand-alone, web-based app
- Explore possibility to integrate to enable clinical use in routine workflows

This work is part of a larger project, the UCANCANDU project. The mission of the UCANCANDU project is to transform child health and accelerate the development and application of personalized medicine tools by harnessing the expertise and resources of a world leading trans disciplinary framework.

Introduction

Multiple criteria decision methods (MCDM) are a generic term for structured, explicit approaches to support decisions making when a decision is influenced by multiple criteria, which have conflicting outcomes in terms of the alternative decisions. In the case of Juvenile Idiopathic Arthritis, the decision whether and when to stop treatment with a biological if a child is in remission, is likely influenced by the trade-off between different patient and disease characteristics that influence the risk of recurrence. MCDA is a tool to aid decision-making by explicitly addressing the trade-offs between possible consequences of tapering (stopping) or continuing treatment.

**Objective:**	**To develop a decision support tool for clinicians to assist in the decision to stop treatment with biologics in children with rheumatoid arthritis.**
**Type of Decision**	Categorical or Understanding Value; Repeated Decision
**Alternatives**	Stop/Taper or Continue Biologics
**Stakeholders**	Clinicians; Patients; Parents
**Required Output**	Overall Value of Taper/Stop or continue Treatment

Table 1. Decision Context

MCDA comprises of a broad set of formal approaches.[1,2] Most have the following steps in common: explicitly determining the decision context; selecting relevant criteria; determining outcomes (performance); scoring alternatives and weighting criteria; combining scores and weights to estimate the overall value of alternatives, and interpreting results (table 2). These steps can be performed in different sequences, and they can be iterative.[2] In the first step of this project, the decision context and desired outcomes were determined with the project team (table 1). In the second step, interviews were performed with 12 Dutch pediatric rheumatologists to select the relevant criteria for the MCDA.

Table 2 – Steps in a value measurement MCDA process.	
**Step**	**Description**
Defining the decision problem	Identify objectives, type of decision, alternatives, stakeholders, and output required
Selecting and structuring criteria	Identify criteria relevant for evaluating alternatives
Measuring performance	Gather data about the alternatives' performance on the criteria and summarize this in a "performance matrix"
Scoring alternatives	Elicit stakeholders' preferences for changes within criteria
Weighting criteria	Elicit stakeholders' preferences between criteria
Calculating aggregate scores	Use the alternatives' scores on the criteria and the weights for the criteria to get "total value" by which the alternatives are ranked
Dealing with uncertainty	Perform uncertainty analysis to understand the level of robustness of the MCDA results
Reporting and examination of findings	Interpret the MCDA outputs, including uncertainty analysis, to support decision making

MCDA, multiple criteria decision analysis.

Table 2 taken from Thokala et al., 2016[2]

Methods

Interviews were performed with pediatric rheumatologists working at Academic Hospitals in the Netherlands, who were already involved in the UCANCANDU project. Eighteen rheumatologists were contacted. Interviews were conducted though Microsoft teams, and audio recorded with permission of the participant.

Interview guide

The interview guide consisted of open ended semi-structured questions. The starting point for the first interviews was a set of possible factors that influence stopping or tapering decisions from a previous focus group study among Canadian Pediatric rheumatologists.

First, the rheumatologists were asked about their preferred timing of a decision to stop or taper in a child without any complications, and their preference for stopping or tapering a biological. Then, for each of factors from the list in box 1, the rheumatologists were asked if, and if so how, each influenced their decision to taper or stop a biological. With regard to the how, specific questions were asked about impact on timing of the decision to stop, and the relationship with other factors. After each category of factors, rheumatologists were asked whether they had any additional factors or considerations they would like to add.

Patient characteristics
- *Patient age*
- *JIA subtype*

Patient history:
- *History or joint damage or erosive disease*
- *History of uveitis*
- *History of spine/SI involvement*
- *History of temporomandibular joint (TMJ) involvement*
- *History of flares*
- *History of comorbidities (e.g. Inflammatory Bowel Disease (IBD), psoriasis)*

Disease Control:
- *How challenging it was to achieve remission (e.g. number of medications or time needed to achieve remission)*
- *Time spent in remission*

Health System Factors:
- *Continuity of care and ability to access follow-up care (e.g. geographical limitations)*
- *Accessibility of biologics after decision to stop*

Box 1. Factors that influence a decision to taper or stop a biological drawn from focus groups in the Canadian setting.

Analysis

The interviewer made notes during the interview and additional findings were summarized immediately after the interview. All interviews were recorded to enable more detail to the summery if necessary. The summary was sent to the participant, and in some cases, specific questions were asked about issues that were unclear during or came up after listening to the audio recording. The pediatric rheumatologists were asked to check the findings and add or adapt as needed, and answer additional questions.

The interviews had an iterative approach. Factors or considerations that were added by rheumatologists, were added to the list of possible criteria in

subsequent interviews. In addition, after four interviews, a first visual representation of the decision process and a value tree were drawn up, which was then presented to the rheumatologists during the subsequent interviews. These were then refined every 2-3 interviews.

Results

Respondent Sample

Twelve pediatric rheumatologists agreed to participate to the interviews. Four participants indicated they no longer work in pediatric rheumatology, one declined our invitation and one potential participant did not respond after two reminders to participate.

Treatment path

During the interviews, it became obvious that the treatment pathway of children with JIA differs, and this impacts the timing of decisions that are made by rheumatologists.

After treatment with the biological is started, most children will respond to treatment between six weeks and six months when the biological is effective. Most children without any response to treatment after six months are switched to a different biological. Some children respond very quickly and remain in remission as soon as 3 months after starting treatment. Others children are slow responders, can have some reduction in symptoms after six months and may be in remission by 12 months. If the child shows some reduction in symptoms after 12 months, but is not in remission, the child is often switched to a different biological.

Timing of a decision to stop or taper biological

Without any complicating factors, most participants stop or taper biologicals after six to 12 months of clinical remission. Some participants indicate, that if a child had a fast response to treatment, they are more likely to stop sooner, but never before six months remission.

Decision to tapering or stop biological immediately

Most participants indicate that they prefer to taper the biological. Some indicate they do this according to a fixed schedule, by increasing the time interval between two injections. Others indicate that they taper based “what feels right for this child”, and thus are flexible in how to taper. Few participants indicate they have no preference for stopping or tapering the biological, they rely on the preference of the child because there is no scientific evidence that supports this decision.

There are two hypotheses mentioned related to the decision to stop or taper the biological. Some participants hypothesize that when they taper, or taper slowly,

there is an increased risk of antibody development. Others feel that with tapering, they have increased control over the JIA, as it is possible to increase dosing immediately if the child shows increased symptoms (flare), and tapering also provides information about the minimal doses at which the JIA is under control.

Age of the child

For most participants, age of the child itself does not influence their decision on when to taper or stop the biological. The impact of age of the child is through other criteria, as an older child is more likely to have other outcomes that influence the decision to stop, such as joint damage or earlier flares of the disease after stopping treatment. A younger child has a higher likelihood that they will outgrow the disease

Serious side-effects

Serious side effects are always a reason to stop the biological immediately for all participants. If the child has been in remission for more than 3 months before the serious side-effects occur, some participants are willing to stop and see whether the child stays in remission. If the child is not yet in remission, or only has been in remission for less than 3 to six months, the child is switched to a different biological.

Mild side-effects

According to most participants mild side effects such as skin rash are no reason to stop earlier, as they can be treated. Some participants indicate that they are willing to taper a few (1-2 months) earlier in a child with mild side-effects, especially in children that fear the needle. That way, the frequency of injections is reduced.

Treatment Compliance

All respondents indicate that low compliance to treatment is not a reason for them to taper or stop, but that the child and the parents have implicitly made that decision. Most indicate that low compliance to treatment is a reason for them to enter into a discussion with the child about the importance of the treatment with the biological to ensure disease control, and the risk of a recurrence or flare if they stop to soon. In most children, they feel that this is sufficient to ensure a few more months of compliance to treatment. Some participants also indicate, that usually other factors, such as fear of injection or side effects), often play a role in the choice of the child (or the parents) not to comply to treatment. Usually, there are ways to treat or manage these.

If a child remains non-compliant, most participants indicate that they will let the child know that they do not support the child's choice to stop treatment. How participants go from there differs. One participant indicated that they will

suggest to the child and/or parent that if they don't comply to treatment, they have to find another doctor. Most might not support a child and/or parents decision, but will continue the relationship. Some will increase the frequency of contact with the child to ensure that they will notice if the child flares, especially if they think that the child or parents will not.

Inflammatory Bowel Disease (IBL), uveitis and psoriasis.

In case of comorbidity, the decision to stop or taper is made in consultation with, or primarily by the gastroenterologist, ophthalmologists or dermatologists.

Active IBL is a reason to continue treatment, also if the JIA is in remission. Some participants indicate that IBL should be in remission longer than JIA, before treatment with biologicals is tapered or stopped, however, they are unsure about the guidelines in case of IBL. The total duration of remission for IBL is between 18 and 24 months before treatment is stopped or tapered, but some participants indicate that they feel that when a child also has IBL, treatment with biologicals is rarely stopped at all.

For uveitis, guidelines suggest that the uveitis has to be in remission for 24 months, before treatment with biologicals is stopped or tapered.

For psoriasis, some participants indicate that they feel it is more difficult to get a child with Psoriasis and JIA into remission than a child who only has JIA. Some participants indicate that in case of psoriasis, they taper until skin reaction occurs, and then increase dosing frequency to the previous step in tapering. Others try to manage recurrent symptoms of psoriasis with local treatment.

Positive rheumatoid factor

The impact of a positive rheumatoid factor on the decision to stop or taper differs between participants. Most participants indicate that the presence of positive rheumatoid factor increases the risk of flares in children with JIA on the population level. Some participants hypothesize that longer treatment in children with positive rheumatoid factors will reduce the risk of flares, others indicate that they don't know and therefore will not prolong treatment, as long as there are no other factors that increase

Joint damage

Most participants emphasize that nowadays, joint damage is rare. If it happens, it is usually because a child is diagnosed late, or the severity of JIA was underestimated and the child was undertreated. Joint damage in itself is not a reason to continue treatment if the joint damage occurred previously and is not become more extensive. Ongoing joint damage is a sign of an active disease process, so as long as joint damage occurs, treatment will not be stopped. It is more likely that the child is switched to a different drug. If joint damage has

occurred during this treatment period, and the child is remission, some participants indicate that they want the child to be in remission between six to 12 months longer before treatment with biologicals is stopped or tapered.

The type of joints affected by JIA

During the first interviews, only involvement of the spine, sacroiliac joint and the temporomandibular joint were suggested as possible reasons to prolong treatment. Most participants indicate these joints are not different in terms of their impact on a decision to taper or stop treatment with biologicals. However, as it is more difficult to make a clinical judgment about whether these joints are in remission, participants often performed an additional test (MRI) to check disease activity in these joints. Some participants mentioned that the MRI is more sensitive to picking up disease activity or changes to the joint compared to clinical tests or x-ray, so this results in caution in interpreting the results.

Additionally, some participants mentioned involvement of larger, weight baring joints such as the knees or hips as a reason to prolong treatment until the child has been in remission for at least six months longer.

Type of JIA

The impact of the type of JIA on decisions to stop or continue treatment was hard to flesh out. It was clear that systemic JIA is very different than non-systemic JIA, in terms of treatment and prognosis. Some participants indicated that other than that, type of JIA did not impact their decision making. Others indicated that in children with more severe disease, they were more likely to continue treatment for longer periods of time. Whether the child has a more severe type of JIA, was judged based on a number of factors, and not all were relevant to all participants. Factors that were mentioned were the number of joints involved, higher levels of inflammation at the start of treatment and involvement of certain types of joints (hip, ankle, knee or wrist), or enthesitis indicate a more severe form of JIA.

If they felt that the child had a more severe JIA, they waited an additional six-12 months before they would stop or taper treatment.

Flares during or after previous attempts to stop

For all participants, a flare of the JIA during or after a previous attempt to stop was a reason to prolong treatment next time. They felt that if a child flared one time before, they would wait an additional six to 12 months before they would consider tapering or stopping the biological.

If the child had flared more than one time, they would either not stop treatment anymore, or try to reduce the biological, if the child has been in remission for at

least 24 months, to the lowest possible dose at which no increase in disease activity occurs.

Number of ineffective biologicals

Some participants indicate that they are less likely to stop treatment if the child had one or more treatment periods with a biological that turned out ineffective, because they (and the child and the parents) were happy that finally they had achieved disease control.

Other rheumatologists indicated that the number of biologicals that they had to try does not impact their decision making, because they assume that the same biological will turn out to be effective, if the child would flare. Others said that they felt, that when a child had flared before, it was sometimes more difficult to achieve remission a next time.

Access to biologicals

All respondents indicate that currently, there are no limitations in access to the biologicals in the Netherlands. Biologicals are reimbursed for all children that need them. It is expected to be different in Canada and part of cross-country validation.

Access to health care

All respondents indicate that in general, access to health care is not an issue in the Netherlands. Some exceptions that were mentioned were:

- Refugee children with a temporary permit, whose permit (and usually that of the whole family) depends partly on their need for biologicals.
- Children from the former Dutch colonies, who can fly to the Netherlands once a year to visit the hospital.
- Children with special needs, such as children with Down syndrome or children from disadvantaged backgrounds, in whom a flare of JIA might be less likely detected.

Patient preference and shared decision making

All respondents indicate that personal circumstances of the child, whether they are school exams, sport activities, or holidays are reasons for them to accelerate or postpone a decision to taper or stop. Most participants are willing to make a decision to stop around 1 to 3 months earlier, although never before six months remission, if this is preferred because of personal circumstances. Similarly, most participants are willing to prolong treatment with between six to 12 months. If children or parents don’t want to stop, usually because of fear of flares, some participants indicate that they find it hard to overrule such a preference, while others emphasize that they will not treat endlessly. Costs of the drug are often

mentioned as a reason why stopping at some point, especially if there are no other complicated factors, is inevitable.

Other factors

Some additional factors were mentioned by a one or a few of participants, but were not supported by others.

Conclusions

Most factors which were discussed in the interviews were reasons to prolong or accelerate a decision for the majority of participants. Only the age of the child and compliance to treatment are not included in the model, for reasons discussed previously. Type of JIA, which includes type and number of joints affected, and presence of enthesitis were collapsed into the criterion "disease severity" along with the disease activity at the start of treatment.

Based on the findings of the interviews, two decision models were made. In figure 1, a distinction is made between reasons to stop or taper sooner, and reasons to postpone a decision to stop or taper. In figure 2, the factors that included in the trade-off on when to stop or taper are included.

Serious side-effects are only included in figure 1, as this is a an important driver for immediate clinical decisions without considering other contextual or clinical factors. All participants indicate that they trade with total duration with which the child is in remission, either by reducing or increasing the time they want the child to be remission before they stop or taper treatment. The impact of the factors (or criteria), in other words the extent to which they postpone or accelerate a decision to taper or stop, differs per participant.

In the next step of this project, the impact of the criteria on the decision to stop will be determined using a weight elicitation process based on multi-criteria decision analysis. From the interviews, it became obvious that there is variation among pediatric rheumatologists in when, and based on which arguments, they make a decision to taper or stop a biological in a child with JIA.

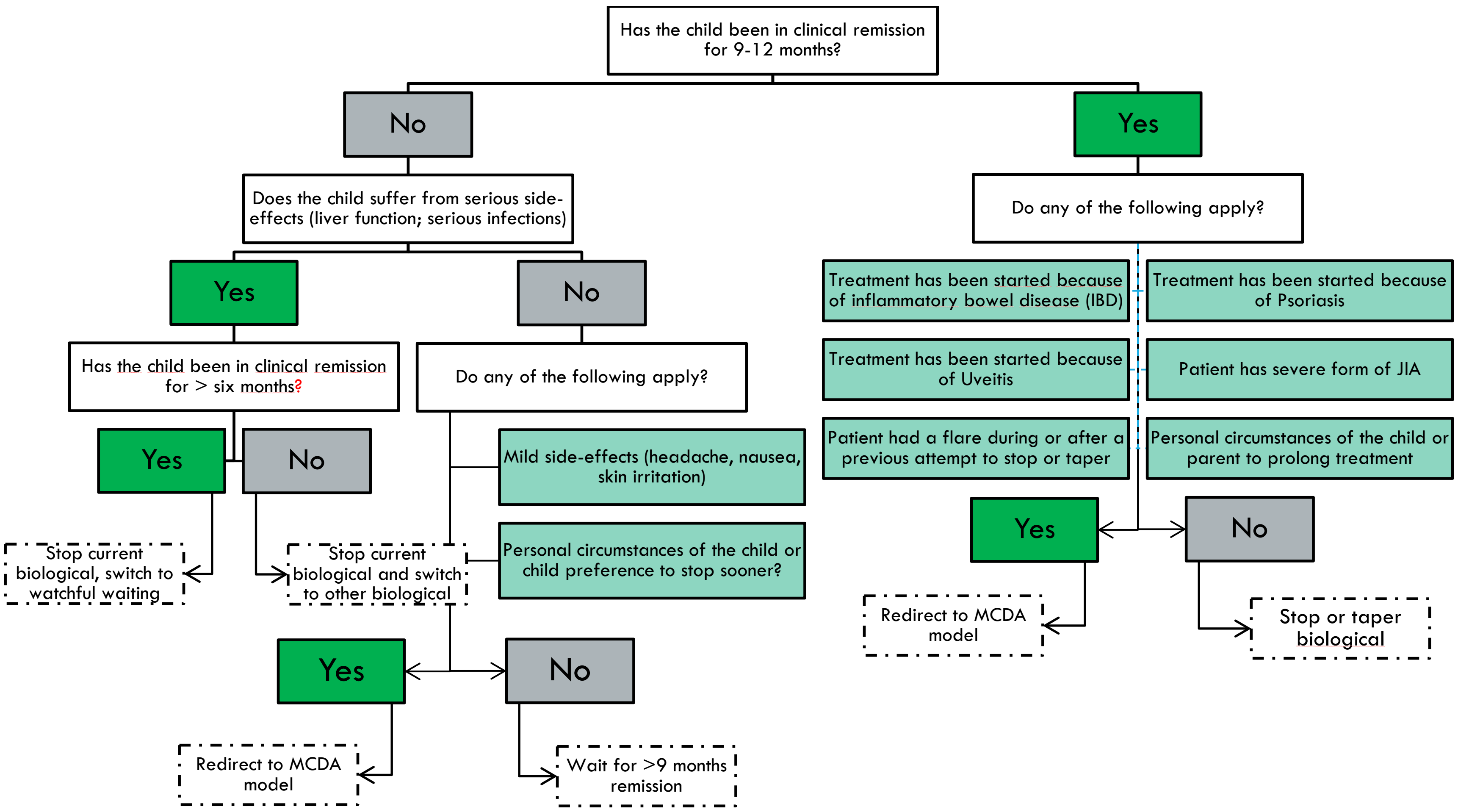

Figure 1. Decision Tree

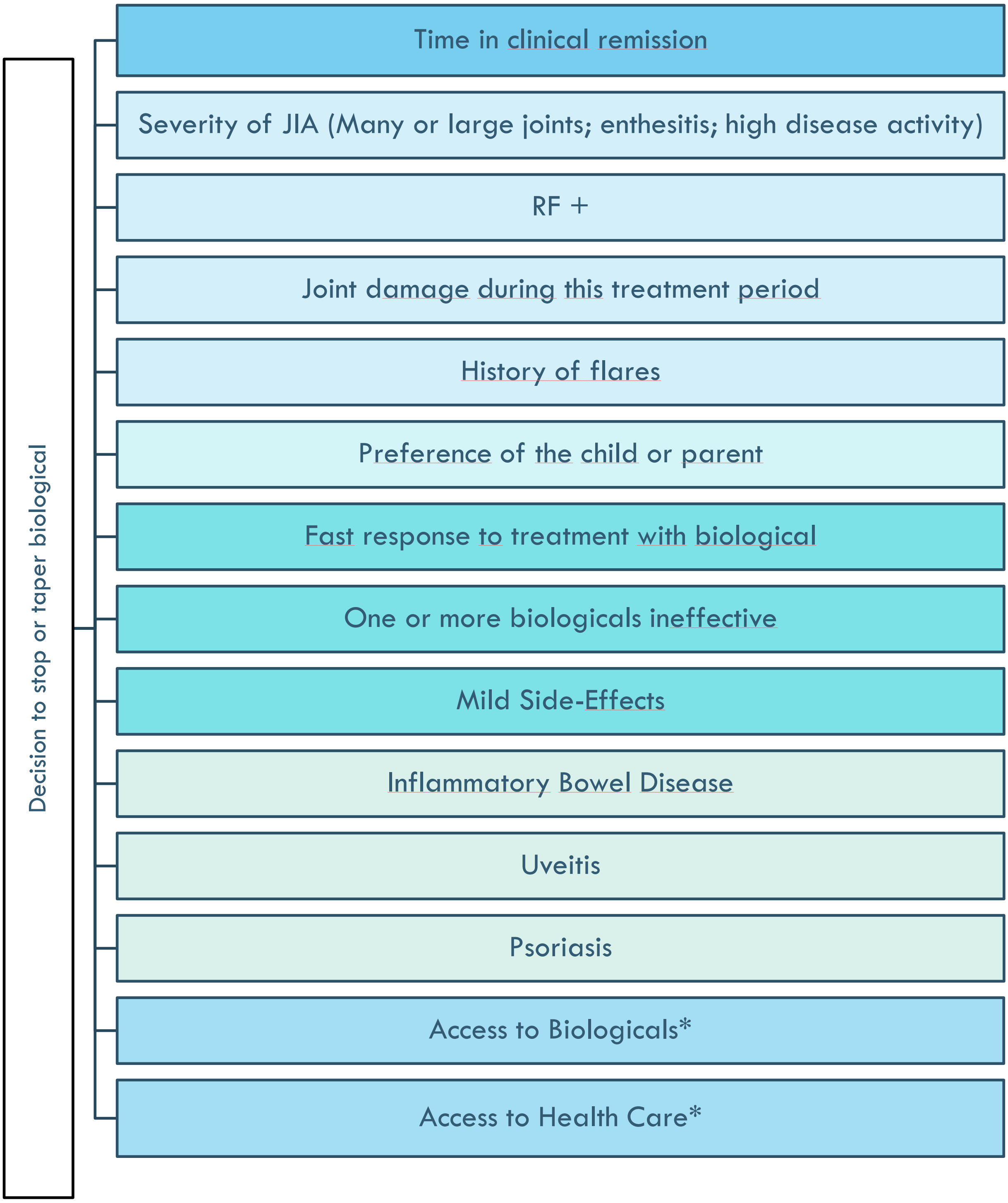

*Figure 2. Draft MCDA model *criteria only relevant in the Canadian setting*
