# Supplementary material for: Withdrawing biologics in non-systemic JIA: what matters to pediatric rheumatologists?
Source: Pediatr Rheumatol Online J. 2023 Jul 11;21:69. doi: 10.1186/s12969-023-00845-4 (PMC10337208; doi:10.1186/s12969-023-00845-4)
Supplement: Supplementary file 2 — Additional file 2: Survey Instrument. [file 12969_2023_845_MOESM2_ESM.docx]

**Supplementary File 2. Survey Instrument**

**UCAN Clinical Vignette Study**

**Introduction**

Dear pediatric rheumatologist,

You are being invited to participate in a survey titled “Juvenile Idiopathic Arthritis: How do pediatric rheumatologists decide when to stop or taper a biological?”.
This survey is being initiated by a team of researchers from the University of Twente in the Netherlands and the University of Calgary in Canada, as part of the UCAN project.

This information sheet is only part of the process of informed consent. It should give you the basic idea of what the research is about and what your participation will involve. If you would like more details about something mentioned here, or information not included here, please ask. Take the time to read this carefully and to understand any accompanying information.

BACKGROUND
There is currently no standard clinical guidance about how physicians should approach decisions about tapering in children with juvenile idiopathic arthritis (JIA) whose disease is in remission. Measuring physician preferences for how best to taper biologic treatment for children with JIA, and in whom, is especially important in the context of therapies for childhood arthritis given the potentially harmful consequences of treatment, the uncertainty around the long-term effects of treatment, and the clinical uncertainty about when and how treatment can be tapered. Lower therapy intensity may reduce the risk of adverse events including infections and malignancies that also contribute to the economic burden associated with additional costly care.

WHAT IS THE PURPOSE OF THE STUDY?
The purpose of this study is gain insight of the current decision process regarding tapering or stopping a biologic in children with juvenile idiopathic arthritis. This is part of an on-going study, and builds on a prior survey that you were invited to participate in and may have completed. The results of this study will be used to develop a tool for pediatric rheumatologists to support this decision.

WHAT WOULD I HAVE TO DO?
You will be asked to complete an online survey and answer questions about tapering decisions you would make in different situations for patients with varying characteristics. It is expected that this will take approximately 15-20 minutes.

WHAT ARE THE RISKS?
There are no anticipated risks associated with participating in this study.

WILL I BENEFIT IF I TAKE PART?
There will be no direct benefits to you from participating in this study. The information we get from this study may help us to better understand what types of patients physicians feel are appropriate to consider tapering off biologic therapy, and this information may contribute to the evidence base to guide tapering choices.

DO I HAVE TO PARTICIPATE?
Participation in the study is completely voluntary. You may withdraw at any point up until the submission of your online survey. If you wish to withdraw while you are in the process of completing the survey, you can choose not to submit it, or submit your responses up until that point. Once submitted, your responses are anonymous and your data can not be withdrawn.

WILL I BE PAID FOR PARTICIPATING, OR DO I HAVE TO PAY FOR ANYTHING?
There are no expenses anticipated with your participation in the study.

WILL MY RECORDS BE KEPT PRIVATE?
Your responses are anonymous and no identifying personal information is being collected. Data collected as part of this research project will be kept confidential. It will only be accessed by the research team members for research purposes. Your confidentiality will be protected at all times during and after the study.

AGREEMENT TO PARTICIPATE
Your decision to complete and return this survey will be interpreted as an indication of your agreement to participate. In no way does this waive your legal rights nor release the investigators, or involved institutions from their legal and professional responsibilities. You are free to withdraw from the study at any time. If you have further questions concerning matters related to this research, please contact: Dr. Gillian Currie Phone: 403-210-6520 or Email: [currie@ucalgary.ca](mailto:currie@ucalgary.ca)

If you have any questions concerning your rights as a possible participant in this research, please contact the Chair of the Conjoint Health Research Ethics Board, Research Services, University of Calgary, 403-220-7990.

The University of Calgary Conjoint Health Research Ethics Board has approved this research study (REB19-0360).

Please select one of the following options below. If you consent to the study you will be taken directly to the survey. If you do not wish to participate in the study, you will be directed to the exit page.

- Yes, I consent to participate in the study (1)
- No, I do not consent to participate in the study (2)

**Q1.3 This survey consists of three parts.**

In the first part, we collect some background information about you as a participant.
In the second part, we use a clinical vignette format to ask you about tapering decisions in different situations. A clinical vignette is a description of a patient using the most important patient characteristics. It is often used in research to understand clinical reasoning.
In the third and final part of this survey, we ask some additional questions related to factors that were not included in the clinical vignette, but could influence your decision making.

**End of Block: Introduction**

**Start of Block: Declined Participation**

*Question:*

Q2.1 You have indicated that you will not participate in the survey. To check whether people that are willing to participate are different from those that are not, we would like to ask you three short questions (age, sex, reason for not participating). Would you be willing to answer these three questions?

- Yes (1)
- No (2)

Q2.2
What is your sex?

- Male (1)
- Female (2)
- Non-binary / third gender (3)
- Prefer not to say (4)

Q2.3 What is your age?

- ≤30 (1)
- 31-40 (2)
- 41-50 (3)
- >50 (4)

Q2.4
What is the main reason to decline participation in this study?

- I am not a pediatric rheumatologist (1)
- I do not have the time to participate (2)
- I am not interested in the topic (3)
- I am not familiar with the topic (4)
- Other, namely (5) __________________________________________________

*Skip To: End of Survey If What is the main reason to decline participation in this study? , I am not a pediatric rheumatologist Is Displayed*

**End of Block: Declined Participation**

**Start of Block: Background Questions**

Q3.1
Part 1. Background characteristics
 What is your sex?

- Male (1)
- Female (2)
- Non-binary / third gender (3)
- Prefer not to say (4)

Q3.2 What is your age?

- ≤30 (1)
- 31-40 (2)
- 41-50 (3)
- >50 (4)

Q3.3 In which country do you practice medicine?

- Canada (1)
- the Netherlands (2)
- Other, (3) __________________________________________________

*Display This Question:*

*If In which country do you practice medicine? = Canada*

Q3.4 In what province/territory do you practice medicine?

- Alberta (1)
- British Columbia (2)
- Manitoba (3)
- New Brunswick (4)
- Newfoundland and Labrador (5)
- Northwest Territories (6)
- Nova Scotia (7)
- Nunavut (8)
- Ontario (9)
- Prince Edward Island (10)
- Quebec (11)
- Saskatchewan (12)
- Yukon (13)

Q3.5 What is your primary practice setting?

- Academic setting, university based (1)
- Academic appointment but community-based practice (2)
- Solo community-based private practice (3)
- Group community-based private practice (4)
- Other (5) __________________________________________________

Q3.6 How many years have you been in practice since training?

- ≤5 years (1)
- 6-10 years (2)
- 11-20 years (3)
- 21-30 years (4)
- >30 years (5)

Q3.7 What percentage of your time do you allocate to clinical work?

________________________________________________________________

Q3.8 Approximately how many **new** JIA patients do you see in a month?

________________________________________________________________

Q3.9 Approximately how many **follow-up** JIA patients do you see in a month?

________________________________________________________________

**End of Block: Background Questions**

**Start of Block: Clinical Vignette Study**

Q4.1
Part 2. Clinical Vignette Study

Imagine you are treating a child diagnosed with non-systemic, RF negative JIA. The child does not have uveitis, IBD or Psoriasis. There is no joint damage, spine or TMJ involvement.


Treatment with MTX was unsuccessful and you want to put the child on their first biologic.

Which biologic would you most likely prescribe?

- Anti-TNF-alfa; adalimumab, etanercept, infliximab (1)
- CTLA-4 fusion protein; abatacept (2)
- Anti-IL-1; anakinra, canakinumab (3)
- Anti-IL-6; tocilizumab (4)
- Anti-CD20; rituximab (5)
- Other, namely (6) __________________________________________________

Q83
The child responds to treatment, does not flare and is in clinical remission on this biologic at six months. Clinical remission means no active arthritis; no fever, no rash, serositis, splenomegaly, or generalized lymphadenopathy attributable to JIA; no active uveitis; normal erythrocyte sedimentation rate or C-reactive protein level (Wallace et al., 2004)

At this time, there are no concerns regarding the ability of the child to access regular and follow-up health care, or to re-start biologics should they need to if an attempt to taper is unsuccessful. Please assume that no other considerations that would make you postpone a decision to taper treatment (either MTX or biologics) apply.

Q84 In your opinion, how long does this child need to be in clinical remission before you would **start to taper MTX**? Select the answer that is closest to your preferred timing. If you use the option to specify a different timing, please specify both number and unit (e.g. 24 months or 2 years).

- 6 months (1)
- 9 months (2)
- 12 months (3)
- 15 months (4)
- 18 months (5)
- Other, namely (6) __________________________________________________
- Never (7)

Q4.3 In your opinion, how long does this child need to be in clinical remission before you would **start to taper the biologic treatment** starting from the moment clinical remission was achieved (assuming the child does not flare after you stopped MTX)? Select the answer that is closest to your preferred timing. If you use the option to specify a different timing, please specify both number and unit (e.g. 24 months or 2 years).

- 6 months (1)
- 9 months (2)
- 12 months (3)
- 15 months (4)
- 18 months (5)
- Other, namely (6) __________________________________________________
- Never (7)

*Skip To: End of Survey If In your opinion, how long does this child need to be in clinical remission before you would start... = Never*

Q4.5 From the moment you start tapering the biologic, how long would it take you to completely stop this biologic if the child does not flare?

- I would not taper but immediately stop (6)
- Less than 6 months (1)
- Between 6 and 12 months (2)
- Between 12 and 18 months (3)
- Between 18 and 24 months (4)
- Other, namely (5) __________________________________________________

Q4.7
In each of the 16 vignettes that are described next, the child's situation is slightly different from the scenario described in the previous section.

Nine patient and treatment characteristics are varied in a systematic way.

The patient and disease characteristics which are varied are:

| **Characteristic** | **Description** | | |
| --- | --- | --- | --- |
| Response to treatment on the current biologic | Child was in remission* at 6 months **or** | Child was in remission* at 12 months |  |
| Rheumatoid factor | Child is RF positive **or** | Child is RF negative |  |
| History of flares** | Child had a flare in the current treatment period **or** | Child has a history of flares in a previous treatment period **or** | Child has no history of flares |
| History of joint damage | Child had joint damage in the current treatment period **or** | Child has no history of joint damage |  |
| History of uveitis | Child had uveitis in the current treatment period, which is in remission **or** | Child has a history of uveitis in a previous treatment period **or** | Child has no history of uveitis |
| Spine involvement | Child had spine involvement in the current treatment period **or** | Child has no history of spine involvement |  |
| TMJ involvement | Child had TMJ involvement in the current treatment period **or** | Child has no history of TMJ involvement |  |
| Patient/Parent preference | Child and parents have a preference to taper the biologic **or** | Child and parents have a preference to continue the biologic |  |
| History of treatment failure with biologics | Child had a treatment failure with a different biologic in the current treatment period **or** | Child has a history of treatment failure with a different biologic in the previous treatment period **or** | Child has no history of treatment failure with a biologic |

*Clinical remission on medication is defined according the Wallace criteria, which include: no active artritis; no fever, no rash, serositis, splenomegaly, or generalized lymphadenopathy attributable to JIA; no active uveitis; normal erythrocyte sedimentation rate or C-reactive protein level (Wallace et al., 2004)
**A flare is defined as any recurrence of disease manifestations after attaining inactive disease

Q4.9

In the following section you will see a series of vignettes, and asked whether you would taper treatment with the current biologic. If you say no, you will be asked to indicate how long the child would need to be remission before you would consider tapering treatment. In this follow up question, you can also decide to taper sooner.

For all vignettes, the patient description is the same, but the characteristics in the table are varied.

*There are no concerns regarding continuity of health care or access to biologics, which means that the child will have access to follow up care and biologics, if an attempt to taper would fail.

Example Question:

A child was diagnosed with non-systemic JIA.

Treatment with MTX was unsuccessful and the child was put on treatment with a biologic.

- The child was in remission at 12 months on the current biologic
- The child is RF Positive
- No history of flares
- No history of joint damage
- Uveitis in the current treatment period, which is in remission
- No history of spine involvement
- TMJ involvement in the current treatment period
- No history of treatment failure with a biologic
- Child and parents prefer to continue the biologic

The child has been in clinical remission (both uveitis and JIA) for [insert answer to Q4.3]

Would you taper the biologic in this child at this time?

O yes
O no

**End of Block: Clinical Vignette Study**

**Start of Block: Vignette 1**

*Display vignette questions only if respondent indicates they are willing to taper biologics.*

Q5.1

A child was diagnosed with non-systemic JIA.

Treatment with MTX was unsuccessful and the child was put on treatment with a biologic.

- The child was in remission at 12 months on the current biologic
- The child is RF Positive
- No history of flares
- No history of joint damage
- No history of uveitis
- No history of spine involvement
- TMJ involvement in the current treatment period
- No history of treatment failure with a biologic
- Child and parents prefer to continue the biologic

The child has been in clinical remission for [insert answer to Q4.3] Would you taper the biologic in this child at this time?

- Yes (1)
- No (2)

Q5.2 Timing

*Display This Question:*

*If 5.1 = No*

Q5.3 How long does this child need to be in clinical remission before you would start tapering the biologic?

- <6 months (1)
- 6-12 months (2)
- 12-18 months (3)
- 18-24 months (4)
- 24-30 months (5)
- 30-36 months (6)
- > 36 months (7)
- I would not taper this child (8)

**End of Block: Vignette 1**

**Start of Block: Vignette 2**

Q6.1

A child was diagnosed with non-systemic JIA.

Treatment with MTX was unsuccessful and the child was put on treatment with a biologic.

- The child was in remission at 6 months on the current biologic
- The child is RF Positive
- History of flares in the previous treatment period
- No history of joint damage
- Uveitis in the current treatment period, which is in remission
- No spine involvement
- No history of TMJ involvement
- Failure of a different biologic in the current treatment period
- Child and parents prefer to continue the biologic

The child has been in clinical remission (both uveitis and JIA) for [insert answer to Q4.3]

Would you taper the biologic in this child at this time?

- Yes (1)
- No (2)

Q6.2 Timing

*Display This Question:*

*If 6.1 = No*

Q6.3 How long does this child need to be in clinical remission before you would start tapering the biologic?

- <6 months (1)
- 6-12 months (2)
- 12-18 months (3)
- 18-24 months (4)
- 24-30 months (5)
- 30-36 months (6)
- > 36 months (7)
- I would not taper this child (8)

**End of Block: Vignette 2**

**Start of Block: Vignette 3**

Q7.1

A child was diagnosed with non-systemic JIA.

Treatment with MTX was unsuccessful and the child was put on treatment with a biologic.

- The child was in remission at 6 months on the current biologic
- The child is RF Positive
- Flare in the current treatment period
- No history of joint damage
- No history of uveitis
- Spine involvement in the current treatment period
- No history of TMJ involvement
- No history of treatment failure with a biologic
- Child and parents prefer to taper the biologic

The child has been in clinical remission for [insert answer to Q4.3].

Would you taper the biologic in this child at this time?

- Yes (1)
- No (2)

Q7.2 Timing

*Display This Question:*

*If 7.1 = No*

Q7.3 How long does this child need to be in clinical remission before you would start tapering the biologic?

- <6 months (1)
- 6-12 months (2)
- 12-18 months (3)
- 18-24 months (4)
- 24-30 months (5)
- 30-36 months (6)
- > 36 months (7)
- I would not taper this child (8)

**End of Block: Vignette 3**

**Start of Block: Vignette 4**

Q8.1

A child was diagnosed with non-systemic JIA.

Treatment with MTX was unsuccessful and the child was put on treatment with a biologic.

- The child was in remission at 12 months on the current biologic
- The child is RF Positive
- No history of flares
- No history of joint damage
- History of uveitis in the previous treatment period
- Spine involvement in the current treatment period
- TMJ involvement in the current treatment period
- Treatment failure with a different biologic in the current treatment period
- Child and parents prefer to taper the biologic

The child has been in clinical remission for [insert answer to Q4.3]

Would you taper the biologic in this child at this time?

- Yes (1)
- No (2)

Q8.2 Timing

*Display This Question:*

*If 8.1 = No*

Q8.3 How long does this child need to be in clinical remission before you would start tapering the biologic?

- <6 months (1)
- 6-12 months (2)
- 12-18 months (3)
- 18-24 months (4)
- 24-30 months (5)
- 30-36 months (6)
- > 36 months (7)
- I would not taper this child (8)

**End of Block: Vignette 4**

**Start of Block: Vignette 5**

Q9.1

A child was diagnosed with non-systemic JIA.

Treatment with MTX was unsuccessful and the child was put on treatment with a biologic.

- The child was in remission at 6 months on the current biologic
- The child is RF Positive
- No history of flares
- Joint damage in the current treatment period
- Uveitis in the current treatment period, which is in remission
- Spine involvement in the current treatment period
- TMJ involvement in the current treatment period
- No history of treatment failure with a biologic
- Child and parents prefer to taper the biologic

The child has been in clinical remission (both uveitis and JIA) for [insert answer to Q4.3]

Would you taper the biologic in this child at this time?

- Yes (1)
- No (2)

Q9.2 Timing

*Display This Question:*

*If 9.1 = No*

Q9.3 How long does this child need to be in clinical remission before you would start tapering the biologic?

- <6 months (1)
- 6-12 months (2)
- 12-18 months (3)
- 18-24 months (4)
- 24-30 months (5)
- 30-36 months (6)
- > 36 months (7)
- I would not taper this child (8)

**End of Block: Vignette 5**

**Start of Block: Vignette 6**

Q10.1

A child was diagnosed with non-systemic JIA.

Treatment with MTX was unsuccessful and the child was put on treatment with a biologic.

- The child was in remission at 6 months on the current biologic
- The child is RF Negative
- No history of flares
- No history of joint damage
- History of uveitis in the previous treatment period
- Spine involvement in the current treatment period
- No history of TMJ involvement
- Treatment failure with a different biologic in the previous treatment period
- Child and parents prefer to continue the biologic

The child has been in clinical remission for [insert answer to Q4.3]

Would you taper the biologic in this child at this time?

- Yes (1)
- No (2)

Q10.2 Timing

*Display This Question:*

*If 10.1 = No*

Q10.3 How long does this child need to be in clinical remission before you would start tapering the biologic?

- <6 months (1)
- 6-12 months (2)
- 12-18 months (3)
- 18-24 months (4)
- 24-30 months (5)
- 30-36 months (6)
- > 36 months (7)
- I would not taper this child (8)

**End of Block: Vignette 6**

**Start of Block: Vignette 7**

Q11.1

A child was diagnosed with non-systemic JIA.

Treatment with MTX was unsuccessful and the child was put on treatment with a biologic.

- The child was in remission at 6 months on the current biologic
- The child is RF Positive
- No history of flares
- Joint damage in the current treatment period
- No history of uveitis
- No history of spine involvement
- TMJ involvement in the current treatment period
- Treatment failure with a different biologic in the previous treatment period
- Child and parents prefer to continue the biologic

The child has been in clinical remission for [insert answer to Q4.3].

Would you taper the biologic in this child at this time?

- Yes (1)
- No (2)

Q11.2 Timing

*Display This Question:*

*If 11.1 = No*

Q11.3 How long does this child need to be in clinical remission before you would start tapering the biologic?

- <6 months (1)
- 6-12 months (2)
- 12-18 months (3)
- 18-24 months (4)
- 24-30 months (5)
- 30-36 months (6)
- > 36 months (7)
- I would not taper this child (8)

**End of Block: Vignette 7**

**Start of Block: Vignette 8**

Q12.1

A child was diagnosed with non-systemic JIA.

Treatment with MTX was unsuccessful and the child was put on treatment with a biologic.

- The child was in remission at 12 months on the current biologic
- The child is RF Positive
- Flare in the current treatment period
- Joint damage in the current treatment period
- History of uveitis in the previous treatment period
- No history of spine involvement
- No history of TMJ involvement
- No history of treatment failure with a biologic
- Child and parents prefer to continue the biologic

The child has been in clinical remission for [insert answer to Q4.3]

Would you taper the biologic in this child at this time?

- Yes (1)
- No (2)

Q12.2 Timing

*Display This Question:*

*If 12.1. = No*

Q12.3 How long does this child need to be in clinical remission before you would start tapering the biologic?

- <6 months (1)
- 6-12 months (2)
- 12-18 months (3)
- 18-24 months (4)
- 24-30 months (5)
- 30-36 months (6)
- > 36 months (7)
- I would not taper this child (8)

**End of Block: Vignette 8**

**Start of Block: Vignette 9**

Q13.1

 A child was diagnosed with non-systemic JIA.

Treatment with MTX was unsuccessful and the child was put on treatment with a biologic.

- The child was in remission at 6 months on the current biologic
- The child is RF Negative
- History of flares in the previous treatment period
- Joint damage in the current treatment period
- History of uveitis in the previous treatment period
- No history of spine involvement
- TMJ involvement in the current treatment period
- No history of treatment failure with a biologic
- Child and parents prefer to taper the biologic

The child has been in clinical remission for [insert answer to Q4.3].

Would you taper the biologic in this child at this time?

- Yes (1)
- No (2)

Q13.2 Timing

*Display This Question:*

*If 13.1 = No*

Q13.3 How long does this child need to be in clinical remission before you would start tapering the biologic?

- <6 months (1)
- 6-12 months (2)
- 12-18 months (3)
- 18-24 months (4)
- 24-30 months (5)
- 30-36 months (6)
- >36 months (7)
- I would not taper this child (8)

**End of Block: Vignette 9**

**Start of Block: Vignette 10**

Q14.1

A child was diagnosed with non-systemic JIA.

Treatment with MTX was unsuccessful and the child was put on treatment with a biologic.

- Treatment failure with a different biologic in the previous treatment period
- The child was in remission at 12 months on the current biologic
- The child is RF Positive
- History of flares in the previous treatment period
- Joint damage in the current treatment period
- No history of uveitis
- Spine involvement in the current treatment period
- No history of TMJ involvement
- Treatment failure with a different biologic in the previous treatment period
- Child and parents prefer to taper the biologic

The child has been in clinical remission for [insert answer to Q4.3]

Would you taper the biologic in this child at this time?

- Yes (1)
- No (2)

Q14.2 Timing

*Display This Question:*

*If 14.1 = No*

Q14.3 How long does this child need to be in clinical remission before you would start tapering the biologic?

- <6 months (1)
- 6-12 months (2)
- 12-18 months (3)
- 18-24 months (4)
- 24-30 months (5)
- 30-36 months (6)
- <36 months (7)
- I would not taper this child (8)

**End of Block: Vignette 10**

**Start of Block: Vignette 11**

Q15.1

A child was diagnosed with non-systemic JIA.

Treatment with MTX was unsuccessful and the child was put on treatment with a biologic.

- The child was in remission at 12 months on the current biologic
- The child is RF Negative
- Flare in the current treatment period
- No history of joint damage
- Uveitis in the current treatment period, which is in remission
- No history of spine involvement
- TMJ involvement in the current treatment period
- Treatment failure with a different biologic in the previous treatment period
- Child and parents prefer to taper the biologic

The child has been in clinical remission for [insert answer to Q4.3]

Would you taper the biologic in this child at this time?

- Yes (1)
- No (2)

Q15.2 Timing

*Display This Question:*

*If 15.1 = No*

Q15.3 How long does this child need to be in clinical remission before you would start tapering the biologic?

- <6 months (1)
- 6-12 months (2)
- 12-18 months (3)
- 18-24 months (4)
- 24-30 months (5)
- 30-36 months (6)
- > 36 months (7)
- I would not taper this child (8)

**End of Block: Vignette 11**

**Start of Block: Vignette 12**

Q16.1

A child was diagnosed with non-systemic JIA.

Treatment with MTX was unsuccessful and the child was put on treatment with a biologic.

- The child was in remission at 12 months on the current biologic
- The child is RF Negative
- No history of flares
- Joint damage in the current treatment period
- No history of uveitis
- No history of spine involvement
- No history of TMJ involvement
- Treatment failure with a different biologic in the current treatment period
- Child and parents prefer to taper the biologic

The child has been in clinical remission for [insert answer to Q4.3].

Would you taper the biologic in this child at this time?

- Yes (1)
- No (2)

Q16.2 Timing

*Display This Question:*

*If 16.1 = No*

Q16.3 How long does this child need to be in clinical remission before you would start tapering the biologic?

- <6 months (1)
- 6-12 months (2)
- 12-18 months (3)
- 18-24 months (4)
- 24-30 months (5)
- 30-36 months (6)
- >36 months (7)
- I would not taper this child (8)

**End of Block: Vignette 12**

**Start of Block: Vignette 13**

Q17.1

A child was diagnosed with non-systemic JIA.

Treatment with MTX was unsuccessful and the child was put on treatment with a biologic.

- The child was in remission at 12 months on the current biologic
- The child is RF Negative
- No history of flares
- Joint damage in the current treatment period
- Uveitis in the current treatment period, which is in remission
- Spine involvement in the current treatment period
- No history of TMJ involvement
- No history of treatment failure with a biologic
- Child and parents prefer to continue the biologic

The child has been in clinical remission (both uveitis and JIA) for [insert answer to Q4.3]

Would you taper the biologic in this child at this time?

- Yes (1)
- No (2)

Q17.2 Timing

*Display This Question:*

*If A17.1 = No*

Q17.3 How long does this child need to be in clinical remission before you would start tapering the biologic?

- <6 months (1)
- 6-12 months (2)
- 12-18 months (3)
- 18-24 months (4)
- 24-30 months (5)
- 30-36 months (6)
- > 36 months (7)
- I would not taper this child (8)

**End of Block: Vignette 13**

**Start of Block: Vignette 14**

Q18.1

A child was diagnosed with non-systemic JIA.

Treatment with MTX was unsuccessful and the child was put on treatment with a biologic.

- The child was in remission at 6 months on the current biologic
- The child is RF Negative
- Flare in the current treatment period
- Joint damage in the current treatment period
- No history of uveitis
- Spine involvement in the current treatment period
- TMJ involvement in the current treatment period
- Failure of a different biologic in the current treatment period
- Child and parents prefer to continue the biologic

The child has been in clinical remission for [insert answer to Q4.3].

Would you taper the biologic in this child at this time?

- Yes (1)
- No (2)

Q18.2 Timing

*Display This Question:*

*If 18.1 = No*

Q18.3 How long does this child need to be in clinical remission before you would start tapering the biologic?

- <6 months (1)
- 6-12 months (2)
- 12-18 months (3)
- 18-24 months (4)
- 24-30 months (5)
- 30-36 months (6)
- > 36 months (7)
- I would not taper this child (8)

**End of Block: Vignette 14**

**Start of Block: Vignette 15**

Q19.1

A child was diagnosed with non-systemic JIA.

Treatment with MTX was unsuccessful and the child was put on treatment with a biologic.

- The child was in remission at 12 months on the current biologic
- The child is RF Negative
- History of flares in the previous treatment period
- No history of joint damage
- No history of uveitis
- Spine involvement in the current treatment period
- TMJ involvement in the current treatment period
- No history of treatment failure with a biologic
- Child and parents prefer to continue the biologic

The child has been in clinical remission for [insert answer to Q4.3].

Would you taper the biologic in this child at this time?

- Yes (1)
- No (2)

Q19.2 Timing

*Display This Question:*

*If 19.1. = No*

Q19.3 How long does this child need to be in clinical remission before you would start tapering the biologic?

- <6 months (1)
- 6-12 months (2)
- 12-18 months (3)
- 18-24 months (4)
- 24-30 months (5)
- 30-36 months (6)
- > 36 months (8)
- I would not taper this child (7)

**End of Block: Vignette 15**

**Start of Block: Vignette 16**

Q20.1

A child was diagnosed with non-systemic JIA.

Treatment with MTX was unsuccessful and the child was put on treatment with a biologic.

- The child was in remission at 6 months on the current biologic
- The child is RF Negative
- No history of flares
- No history of joint damage
- No history of uveitis
- No history of spine involvement
- No history of TMJ involvement
- No history of treatment failure with a biologic
- Child and parents prefer to taper the biologic

The child has been in clinical remission since [insert answer to Q4.3].

Would you taper the biologic in this child at this time?

- Yes (1)
- No (2)

Q20.2 Timing

*Display This Question:*

*If A20.1 = No*

Q20.3 How long does this child need to be in clinical remission before you would start tapering the biologic?

- <6 months (1)
- 6-12 months (2)
- 12-18 months (3)
- 18-24 months (4)
- 24-30 months (5)
- 30-36 months (6)
- > 36 months (8)
- I would not taper this child (7)

**End of Block: Vignette 16**

**Start of Block: Additional Questions**

Q21.1
You have answered the last of the 16 clinical vignettes.

In these vignettes, there were some children in which the first biologic failed.

Imagine this happens in a child diagnosed with non-systemic, RF negative JIA. The child does not have uveitis, IBD or Psoriasis. There is no joint damage, spine or TMJ involvement.

Treatment with MTX was unsuccessful and there is a failure of treatment with your first choice of biologic, which was [insert answer Q4.1] (this is the answer you gave when you were asked about your first choice of treatment in this child).

If this were the case, what would be your second choice of biologic?

- Anti-TNF-alfa; adalimumab, etanercept, infliximab (1)
- CTLA-4 fusion protein; abatacept (2)
- Anti-IL-1; anakinra, canakinumab (3)
- Anti-IL-6; tocilizumab (4)
- Anti-CD20; rituximab (5)
- Other, namely (6) __________________________________________________

Q21.2
Part 3. Other considerations

In this last part of the survey, we have questions about additional considerations which might play a role in a decision on when to taper.

Again, consider the situation in which you are treating a child diagnosed with non-systemic, RF negative JIA.
The child does or did not have uveitis, IBD or Psoriasis.
There was no joint damage, spine or TMJ involvement.
Treatment with MTX was unsuccessfull.

The child is in remission at 6 months on the current biologic.

What would be the effect of the following considerations on your decision to taper the biologic?

If there are other factors, which were not mentioned before, but do influence the timing of your decision to taper, you can add them to this list.

|  | Taper sooner (1) | Taper at [insert answer Q4.3] (2) | Taper later (3) |
| --- | --- | --- | --- |
| Pain or swelling at the injection site (3) |  |  |  |
| Fear of injections (16) |  |  |  |
| Polyarticular onset of disease (4) |  |  |  |
| Enthesitis (5) |  |  |  |
| Asymmetric joint involvement (6) |  |  |  |
| Higher disease activity at the start of treatment with biologics (7) |  |  |  |
| Involvement of the ankle joint (8) |  |  |  |
| Involvement of the sacro-iliac joint (9) |  |  |  |
| Involvement of the finger or toe joints (10) |  |  |  |
| Involvement of the hip joint (11) |  |  |  |
| Involvement of the knee joint (17) |  |  |  |
| Other, namely (13) |  |  |  |
| Other, namely (14) |  |  |  |
| Other, namely (15) |  |  |  |

*Display This Question only in respondents from Canada*

Q21.3 What would be the effect of the following considerations on your decision to taper the biological?

|  | Taper sooner (1) | Taper at [insert answer Q4.3] (2) | Taper later (3) |
| --- | --- | --- | --- |
| Difficulty for patients/parents accessing my clinical practice due to distance or weather conditions (1) |  |  |  |
| Difficulty accessing the biologic again once it is stopped due to insurance restrictions (2) |  |  |  |

*Display This Question only in respondents from Canada*

Q21.4 What percentage of your patients has trouble accessing your clinical practice for their JIA as a result of distance to practice and/or weather conditions during winter?

- <25%
- 25-50% (2)
- 50-75% (3)
- >75% (4)

*Display This Question only in respondents from Canada*

Q21.5 What percentage of your patients has trouble accessing biologics due to insurance restrictions again after their treatment with biologics has stopped?

- 25%
- 25-50% (2)
- 50-75% (3)
- >75% (4)

Q21.6 You have reached the end of this survey. You can use the text box below to give any additional input to our research team. This includes any assumptions you made while answering the clinical vignettes because in your opinion, information was missing from the clinical vignettes.

________________________________________________________________

________________________________________________________________

________________________________________________________________

________________________________________________________________

________________________________________________________________

**End of Block: Additional Questions**
