# Supplementary material for: Withdrawing biologics in non-systemic JIA: what matters to pediatric rheumatologists?
Source: Pediatr Rheumatol Online J. 2023 Jul 11;21:69. doi: 10.1186/s12969-023-00845-4 (PMC10337208; doi:10.1186/s12969-023-00845-4)
Supplement: Supplementary file 3 — Additional file 3: Supplementary Table 1. Characteristics and levels inluded in the clinical vignettes. [file 12969_2023_845_MOESM3_ESM.docx]

**Supplementary Table 1.** The patient, treatment and disease characteristics with the different outcomes as they were varied in the clinical vignette.

| **Characteristic** |  | **Possible outcomes** |
| --- | --- | --- |
| Response to treatment on the current biologic | 1 | Child was in remission* at 12 months |
|  | 2 | Child was in remission* at 6 months |
| Rheumatoid factor | 1 | Child is rheumatoid factor positive |
|  | 2 | Child is rheumatoid factor negative |
| History of flares** | 1 | Child had a flare in the current treatment period |
|  | 2 | Child has a history of flares in a previous treatment period |
|  | 3 | Child has no history of flares |
| History of joint damage | 1 | Child had joint damage in the current treatment period |
|  | 2 | Child has no history of joint damage |
| History of uveitis*** | 1 | Child had uveitis in the current treatment period, which is in remission |
|  | 2 | Child has a history of uveitis in a previous treatment period |
|  | 3 | Child has no history of uveitis |
| Spine involvement | 1 | Child had spine involvement in the current treatment period |
|  | 2 | Child has no history of spine involvement |
| TMJ involvement | 1 | Child had TMJ involvement in the current treatment period |
|  | 2 | Child has no history of TMJ involvement |
| Patient/Parent preference | 1 | Child and parents have a preference to continue the biologic |
|  | 2 | Child and parents have a preference to withdraw the biologic |
| History of treatment failure with biologics | 1 | Child had a treatment failure with a different biologic in the current treatment period |
|  | 2 | Child has a history of treatment failure with a different biologic in the previous treatment period |
|  | 3 | Child has no history of treatment failure with a biologic |

*Clinical remission on medication is defined according to the Wallace criteria, which include: no active arthritis; no fever, no rash, serositis, splenomegaly, or generalized lymphadenopathy attributable to JIA; no active uveitis; normal erythrocyte sedimentation rate or C-reactive protein level (Wallace et al., 2004)
**A flare is defined as any recurrence of disease manifestations after first attaining clinical remission

*** Uveitis is assumed to be in remission at the same time as the JIA.

TMJ = temporomandibular joint
