# Supplementary material for: Withdrawing biologics in non-systemic JIA: what matters to pediatric rheumatologists?
Source: Pediatr Rheumatol Online J. 2023 Jul 11;21:69. doi: 10.1186/s12969-023-00845-4 (PMC10337208; doi:10.1186/s12969-023-00845-4)
Supplement: Supplementary file 4 — Additional file 4: Supplementary Table 2. The experimental design of the 16 clinical vignettes. [file 12969_2023_845_MOESM4_ESM.docx]

**Supplementary Table 2.** The experimental design of 16 vignettes with the outcomes for all characteristic shown in each vignette

| **Question number** | **Response to treatment on the current biologic** | **Rheumatoid factor** | **History of flares** | **History of joint damage** | **History of uveitis** | **Spine involvement** | **TMJ involvement** | **History of treatment failure with biologics** | **Patient/Parent preference** |
| --- | --- | --- | --- | --- | --- | --- | --- | --- | --- |
| **1** | 2 | 1 | 1 | 1 | 1 | 1 | 2 | 1 | 2 |
| **2** | 1 | 1 | 2 | 1 | 3 | 1 | 1 | 3 | 2 |
| **3** | 1 | 1 | 3 | 1 | 1 | 2 | 1 | 1 | 1 |
| **4** | 2 | 1 | 1 | 1 | 2 | 2 | 2 | 3 | 1 |
| **5** | 1 | 1 | 1 | 2 | 3 | 2 | 2 | 1 | 1 |
| **6** | 1 | 2 | 1 | 1 | 2 | 2 | 1 | 2 | 2 |
| **7** | 1 | 1 | 1 | 2 | 1 | 1 | 2 | 2 | 2 |
| **8** | 2 | 1 | 3 | 2 | 2 | 1 | 1 | 1 | 2 |
| **9** | 1 | 2 | 2 | 2 | 2 | 1 | 2 | 1 | 1 |
| **10** | 2 | 1 | 2 | 2 | 1 | 2 | 1 | 2 | 1 |
| **11** | 2 | 2 | 3 | 1 | 3 | 1 | 2 | 2 | 1 |
| **12** | 2 | 2 | 1 | 2 | 1 | 1 | 1 | 3 | 1 |
| **13** | 2 | 2 | 1 | 2 | 3 | 2 | 1 | 1 | 2 |
| **14** | 1 | 2 | 3 | 2 | 1 | 2 | 2 | 3 | 2 |
| **15** | 2 | 2 | 2 | 1 | 1 | 2 | 2 | 1 | 2 |
| **16** | 1 | 2 | 1 | 1 | 1 | 1 | 1 | 1 | 1 |

TMJ = temporomandibular joint
