# Supplementary material for: Withdrawing biologics in non-systemic JIA: what matters to pediatric rheumatologists?
Source: Pediatr Rheumatol Online J. 2023 Jul 11;21:69. doi: 10.1186/s12969-023-00845-4 (PMC10337208; doi:10.1186/s12969-023-00845-4)
Supplement: Supplementary file 5 — Additional file 5: Supplementary Table 3. Descriptive data for each of the 16 clinical vignettes. [file 12969_2023_845_MOESM5_ESM.docx]

**Supplementary Table 3.** Descriptive data for each of the 16 clinical vignettes. In the second column, the number of respondents and the percentage of the total respondent sample that would withdraw biologic therapy at their minimum treatment time in CID. In columns 3 to 10, the number of respondents that would withdraw biologic therapy in each time interval.

|  | | | | | Treatment duration | | | | |  | | |
| --- | --- | --- | --- | --- | --- | --- | --- | --- | --- | --- | --- | --- |
| clinical vignette number | **Witdraw biologic therapy at minimal treatment time* (n, %)** | | **3-6 months** | **6-12 months** | | **12-18 months** | **18-24 months** | **24-30 months** | **30-36 months** | | **>36 months** | **Do not taper** |
| 1 | 9 | 27% | 1 | 3 | | 8 | 8 | 4 | 1 | | 2 | 6 |
| 2 | 2 | 6% | 0 | 2 | | 3 | 5 | 12 | 2 | | 3 | 6 |
| 3 | 13 | 39% | 0 | 6 | | 7 | 12 | 4 | 0 | | 0 | 4 |
| 4 | 5 | 15% | 0 | 4 | | 4 | 13 | 3 | 2 | | 2 | 5 |
| 5 | 6 | 18% | 0 | 2 | | 3 | 10 | 8 | 1 | | 6 | 3 |
| 6 | 4 | 12% | 0 | 2 | | 9 | 11 | 4 | 1 | | 4 | 2 |
| 7 | 4 | 12% | 0 | 2 | | 5 | 7 | 6 | 0 | | 4 | 9 |
| 8 | 1 | 3% | 0 | 2 | | 5 | 6 | 9 | 0 | | 5 | 6 |
| 9 | 14 | 42% | 0 | 3 | | 10 | 9 | 6 | 3 | | 1 | 1 |
| 10 | 6 | 18% | 0 | 2 | | 8 | 8 | 8 | 0 | | 1 | 6 |
| 11 | 4 | 12% | 0 | 3 | | 0 | 13 | 11 | 2 | | 2 | 2 |
| 12 | 14 | 42% | 0 | 4 | | 11 | 13 | 2 | 1 | | 2 | 0 |
| 13 | 2 | 6% | 0 | 2 | | 4 | 9 | 8 | 3 | | 6 | 1 |
| 14 | 1 | 3% | 0 | 3 | | 6 | 8 | 7 | 0 | | 3 | 6 |
| 15 | 6 | 18% | 0 | 4 | | 8 | 10 | 6 | 0 | | 2 | 3 |
| 16 | 30 | 91% | 0 | 8 | | 17 | 8 | 0 | 0 | | 0 | 0 |

*Minimum treatment time in CID is the pediatric answer to the question when pediatric rheumatologists would withdraw treatment in a child without contra-indications. Minimum treatment time varied between 6 and 21 months in CID between respondents.

TMJ = temporomandibular joint
