# Supplementary material for: Withdrawing biologics in non-systemic JIA: what matters to pediatric rheumatologists?
Source: Pediatr Rheumatol Online J. 2023 Jul 11;21:69. doi: 10.1186/s12969-023-00845-4 (PMC10337208; doi:10.1186/s12969-023-00845-4)
Supplement: Supplementary file 6 — Additional file 6: Supplementary Table 4. Withdrawal of biologic therapy at each time interval in response to the clinical vignettes. [file 12969_2023_845_MOESM6_ESM.docx]

**Supplementary Table 4.** The number and % of pediatric rheumatologists that would withdraw biologic therapy at each time interval after achieving clinically inactive disease (CID) in response to the 16 clinical vignettes (group level data)

|  | **Treatment Duration** | | | | | | | |
| --- | --- | --- | --- | --- | --- | --- | --- | --- |
|  | **3-6 months** | **6-12 months** | **12-18 months** | **18-24 months** | **24-30 months** | **30-36 months** | **>36 months** | **Do not taper** |
| **N** | 15 | 75 | 81 | 140 | 98 | 16 | 43 | 60 |
| **%** | 3% | 14% | 15% | 27% | 19% | 3% | 8% | 11% |
