# Supplementary material for: Withdrawing biologics in non-systemic JIA: what matters to pediatric rheumatologists?
Source: Pediatr Rheumatol Online J. 2023 Jul 11;21:69. doi: 10.1186/s12969-023-00845-4 (PMC10337208; doi:10.1186/s12969-023-00845-4)
Supplement: Supplementary file 7 — Additional file 7: Supplementary Table 5. Perceived influence of other disease characteristics on the withdrawal decision. [file 12969_2023_845_MOESM7_ESM.docx]

**Supplementary Table 5.** The disease characteristics that were excluded from the clinical vignette and their perceived influence on withdrawal decisions.

| **Factor** | **Sooner** | **Minimal treatment time** | **Later** | **Missing** |
| --- | --- | --- | --- | --- |
| Hip involvement | 1 | 7 | 19 | 2 |
| Sacroiliac joint involvement | 0 | 10 | 18 | 1 |
| High disease activity | 1 | 13 | 14 | 1 |
| Pain injection site | 10 | 17 | 0 | 2 |
| Fear of injections | 11 | 17 | 0 | 1 |
| Polyarticular onset | 2 | 18 | 8 | 1 |
| Enthesitis | 0 | 19 | 8 | 2 |
| Ankle involvement | 1 | 19 | 8 | 1 |
| Assymetric joint involvement | 2 | 23 | 1 | 3 |
| Finger and toe involvement | 1 | 24 | 2 | 2 |
| Knee involvement | 3 | 24 | 0 | 2 |

In an additional question in the survey, respondents were asked to indicate, for each of the factors, whether they would taper sooner, later or at their minimal treatment time for each of the factors.
