# Supplementary material for: Withdrawing biologics in non-systemic JIA: what matters to pediatric rheumatologists?
Source: Pediatr Rheumatol Online J. 2023 Jul 11;21:69. doi: 10.1186/s12969-023-00845-4 (PMC10337208; doi:10.1186/s12969-023-00845-4)
Supplement: Supplementary file 8 — Additional file 8: Supplementary Table 6. Decision to continue biologic therapy dependent upon country. Supplementary Table 7. Treatment duration with biologic therapy dependent upon country. [file 12969_2023_845_MOESM8_ESM.docx]

**Supplementary Tables 6 and 7**

**Supplementary Table 6.** Decision to continue treatment with biologic therapy


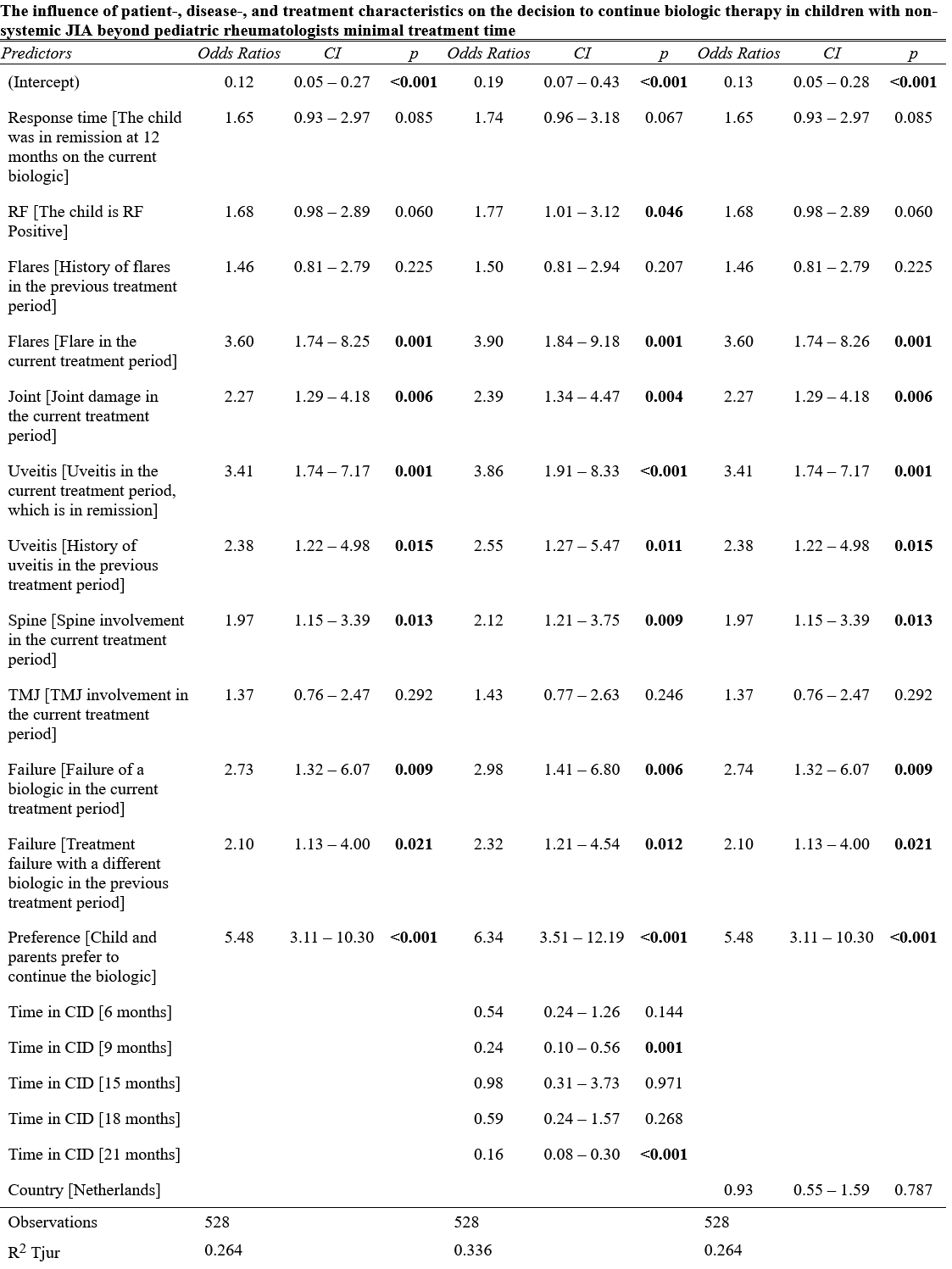


**model 3**

**model 1**

**model 2**

Three models based on logistic regression analysis, with: 1) only the characteristics included in the clinical vignette as independent variables, 2) with country of residence added as a covariate, and 3) with minimal treatment time added as a covariate. The dependent variable is the decision to taper (0) or continue (1) biologic therapy. CID = clinically inactive disease; JIA = juvenile idiopathic arthritis; RF = rheumatoid factor; TMJ = temporomandibular joint.

**Supplementary Table 7.** Treatment duration with biologic therapy


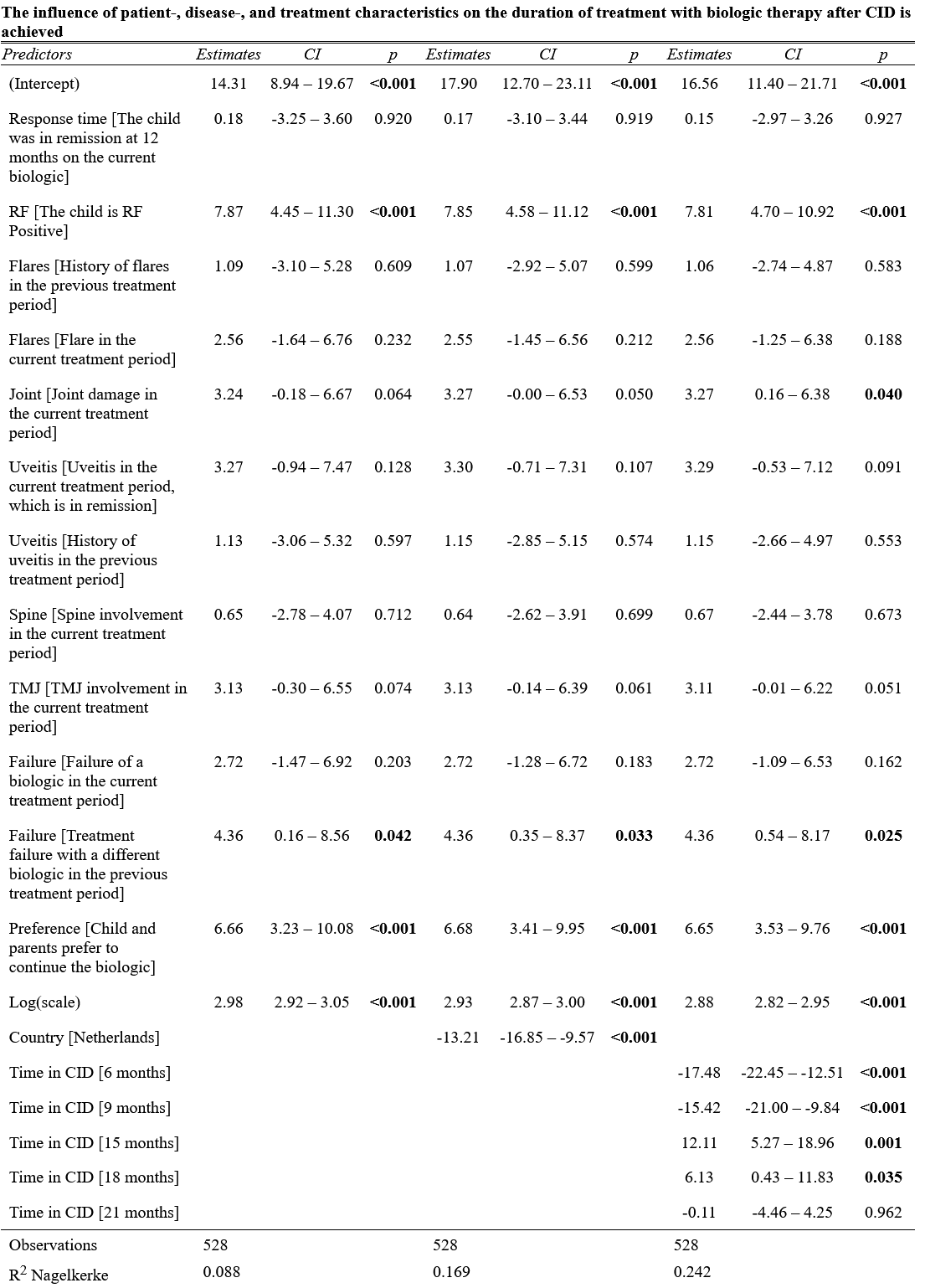


**model 3**

**model 2**

**model 1**

Three models for the interval regression analysis, with: 1) only the characteristics included in the clinical vignette as independent variables, 2) with country of residence as a covariate, and 3) with minimal treatment time as a covariate. The dependent variable is the total treatment duration after clinically inactive disease is achieved (6-month intervals between 6 and 48 months). CID = clinically inactive disease; JIA = juvenile idiopathic arthritis; RF = rheumatoid factor; TMJ = temporomandibular joint.
